# Recurrent antibody repertoire features associated with six selected human IGHV genes: V(D)J usage, heavy-light pairing, and CDRH3 properties

**DOI:** 10.3389/fimmu.2026.1888170

**Published:** 2026-09-21

**Authors:** Mengmeng Zhang, Daojing Wang, Wenwen Xi, Huihui Jia, Beifen Shen, Yan Wen, Jing Wang, Jiannan Feng

**Affiliations:** 1 Academy of Military Medical Sciences, Beijing, China; 2 State Key Laboratory of National Security Specially Needed Medicines, Beijing, China; 3 College of Biotechnology, Jiangsu University of Science and Technology, Zhenjiang, China; 4 Joint National Laboratory for Antibody Drug Engineering, The First Affiliated Hospital of Henan University, Henan University, Kaifeng, China

**Keywords:** IGHV genes, V(D)J recombination, VH-VL pairing, CDRH3 physicochemical features, Observed Antibody Space, paired antibody repertoire

## Abstract

**Introduction:**

Several human IGHV genes recur in infection, autoreactivity, B-cell malignancy, and antibody-discovery studies, but their downstream V(D)J usage, observed heavy-light pairing, and CDRH3 properties have rarely been examined together in paired human repertoires.

**Methods:**

We assembled 3,003,127 paired heavy- and light-chain records from public Observed Antibody Space (OAS) source files. After quality control, gene-level standardization, and exact-clonotype collapsing, the primary six-gene analysis included 712,143 exact clonotypes assigned to IGHV1-2, IGHV1-69, IGHV3-21, IGHV3-23, IGHV3-30, or IGHV4-34.

**Results:**

Marginal V-D effects were generally modest, whereas recurrent V-conditioned D-J modules and IGHV-associated VH-VL enrichment or depletion were repeatedly observed across OAS study groups in productive expressed repertoires. Several VH-VL directions persisted across studies and metadata-defined immune contexts, although two pairs were context-sensitive. External comparison with PairedAbNGS supported 8 of 10 evaluable key VH-VL directions and the broad IGHV-associated CDRH3 profiles. IGHV4-34 showed a study-robust, cross-resource positive shift in CDRH3 side-chain charge score.

**Discussion:**

Together, these analyses provide a study-aware reference for V-conditioned D-J usage, VH-VL associations, and CDRH3 properties across six selected IGHV backgrounds, while defining the limits imposed by immune-context heterogeneity and productive-repertoire sampling.

## Introduction

Human antibody diversity is generated by V(D)J recombination, junctional insertion and deletion, and somatic hypermutation (1–4). The expressed repertoire, however, is not a random draw from all possible sequences. It reflects recombination accessibility, productive selection, clonal expansion, antigen exposure, and host background. Paired heavy-light chain sequencing makes it possible to connect these layers within the same antibody molecules by linking heavy-chain rearrangement, light-chain usage, and CDRH3 sequence features.

Antibody repertoires can converge across individuals despite their enormous theoretical diversity. Public clonotypes and stereotyped antibody features have been described in baseline repertoires (5) and in responses to dengue (6), SARS-CoV-2 (7–9), influenza virus (10, 11), HIV-1 (12, 13), and hepatitis C virus (14). Such convergence suggests that the human antibody repertoire contains reproducible organization despite the stochastic nature of diversification. For IGHV genes repeatedly used in immunologically relevant settings, the next question is whether downstream D/J usage, light-chain pairing, and CDRH3 composition also carry gene-linked patterns.

Before the downstream analyses, we selected six IGHV genes: IGHV1-2, IGHV1-69, IGHV3-21, IGHV3-23, IGHV3-30, and IGHV4-34. These genes were chosen because they are well represented in public paired-repertoire data and have been repeatedly implicated in infection-associated responses, autoreactivity, B-cell malignancy, or antibody discovery studies (**Figure 1**). IGHV1–69 has been reported in influenza and HCV responses (10, 15–18), IGHV3-family related genes including IGHV3-30–3 in SARS-CoV-2 responses (19), IGHV3–30 in Plasmodium falciparum responses (20), IGHV1–2 in pathogen-associated responses (21), IGHV4–34 in autoreactive repertoires (22–25), and IGHV3-21, IGHV3-23, or IGHV4–34 in B-cell malignancy contexts (26–31). The selection was biologically motivated and was not intended to represent an unbiased survey of the IGHV locus.

**Figure 1:**
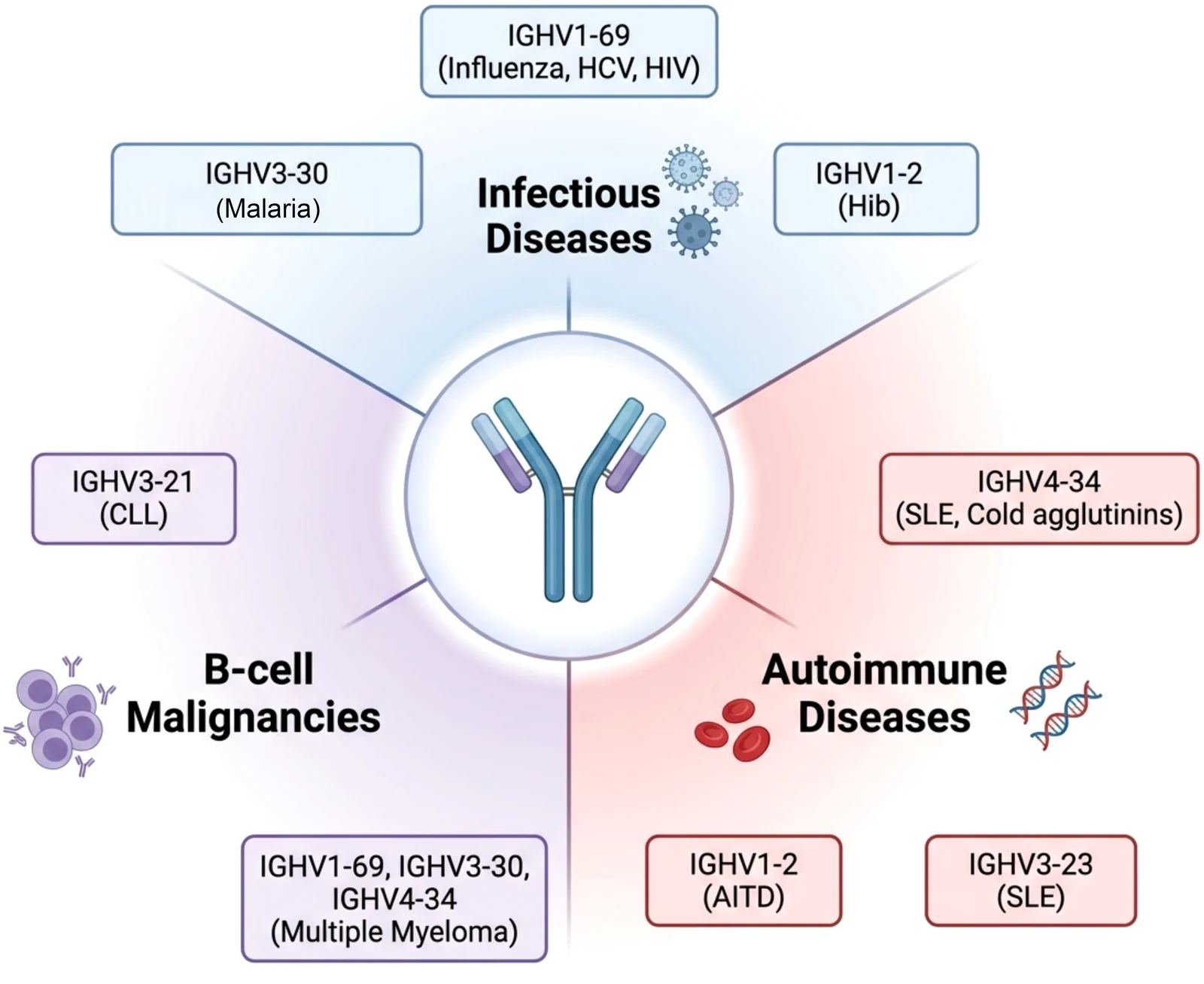
Biological and clinical settings associated with the six selected human IGHV genes. The schematic illustrates the selection of six representative IGHV genes (IGHV1-2, IGHV1-69, IGHV3-21, IGHV3-23, IGHV3-30, and IGHV4-34) and their association with three major disease categories.

These genes are also informative backgrounds for studying repertoire organization. IGHV usage can be shaped by germline copy-number variation, allelic diversity, locus organization and recombination signal features (32–34). Observed frequency is therefore neither a pure measure of antigen-driven selection nor a direct estimate of primary recombination probability. Gene-centered analysis helps distinguish broad IGHV abundance from downstream V-D-J, VH-VL, and CDRH3 patterns that are specific to a heavy-chain context.

OAS provides the scale required for this analysis, but combines studies that differ in donor background, immune state, B-cell subset, isotype, sequencing platform, and sampling depth. Pooled estimates may therefore reflect disease, vaccination, antigen experience, or study composition. We quantified this composition and tested selected associations across studies and disease/vaccine-defined metadata groups. These analyses assess robustness but cannot replace matched naive and antigen-experienced compartments, which were not uniformly identified in the available metadata.

Previous studies have described V-gene usage, public clonotypes, generation probabilities, antibody structure, binding mechanisms, and non-random VH-VL pairing (35–53). It remains unclear how these pairing patterns relate to V-linked D-J organization and CDRH3 sequence properties. We therefore analyzed V-D-J usage, VH-VL enrichment, and CDRH3 features for six predefined IGHV backgrounds, together with study-, immune-context-, clonotype-, and cross-resource sensitivity analyses.

## Materials and methods

### Dataset construction

Paired heavy- and light-chain antibody sequences were retrieved from the Observed Antibody Space (OAS) database (https://opig.stats.ox.ac.uk/webapps/oas/), a public repository of annotated antibody repertoire sequencing data (44). OAS integrates next-generation sequencing datasets from multiple sources, including the Sequence Read Archive, and provides standardized V(D)J gene annotation using ANARCI (58) and IgBLAST (59).

All available human paired heavy-light chain datasets were downloaded from OAS as of January 2026. The native paired OAS release comprised 3, 003, 127 records from 580 source files distributed across 19 OAS URL study directories. Of these, 563 source files containing 2, 965, 738 records were mapped to 17 OAS DS Name/source-study groups in the structured metadata table; 17 additional source files containing 37, 389 records lacked a validated study-level mapping and were retained only in the native-source productivity audit. Among the metadata-mapped records, 2, 965, 731 had a valid standardized IGHV assignment. The six focus IGHV genes accounted for 888, 853 sequence records before the final light-chain V-gene eligibility check. Five records lacked an interpretable light-chain V-gene call, leaving 888, 848 records and 712, 143 exact clonotypes for paired-gene analyses; 712, 148 exact-clonotype representatives were eligible for CDRH3-only analyses. Source-file and study-level metadata are summarized in **Supplementary Table 1**. For sensitivity analyses, source files were further grouped from the available disease and vaccine annotations as infectious disease, vaccinated/convalescent, disease plus vaccine, autoimmune/inflammatory, allergy, malignancy, other disease, or unclassified/unannotated. The latter category was retained as a metadata category and was not interpreted as healthy or naive because standardized B-cell subset labels were unavailable.

To assess whether non-productive rearrangements could serve as a comparator, we audited all 580 locally available paired OAS source files using each file’s native header. The structured metadata workbook mapped 563 files to 17 validated OAS DS Name/source-study groups; the remaining 17 files, derived from three OAS URL study directories, were retained in the source-schema audit but were not used for study-stratified analyses. In this paired source release, all rows were annotated as productive, and no non-productive subset was available for productive-versus-non-productive V-D or D-J comparisons.

Records were included if they contained paired heavy and light chains, had clear heavy-chain V, D, and J gene annotations and light-chain V and J annotations, and had heavy- and light-chain CDR3 lengths of 5–30 amino acids according to IMGT numbering (2). Allelic annotations were collapsed to gene level, for example IGHV1-69*01 was treated as IGHV1-69. Records with missing, non-interpretable, or multiple competing heavy-chain V-gene assignments were excluded from the six-IGHV analysis rather than reassigned to a paralogous gene.

In the primary analysis, exact paired clonotypes were defined as sequences sharing the same heavy-chain V gene, heavy-chain J gene, heavy-chain CDRH3 amino acid sequence, light-chain V gene, light-chain J gene, and light-chain CDR3 amino acid sequence; alleles were collapsed to gene level. We use the term “exact clonotype” for this primary definition because it reduces duplicate sequence-level representation but does not necessarily merge all somatic variants within a broader B-cell lineage.

In the primary six-IGHV analysis, we focused on six V genes with established relevance to public antibody responses and disease-associated repertoires: IGHV1-69, IGHV1-2, IGHV3-23, IGHV3-30, IGHV3-21, and IGHV4-34. Together, these genes contributed 888, 848 sequence-level records and 712, 143 exact clonotypes. Unless otherwise specified, downstream V-D, conditional D-J, VH-VL and CDRH3 analyses were performed on exact clonotype within this six-IGHV subset.

For clonotype de-duplication, multiple sequences assigned to the same exact clonotype were collapsed so that one representative sequence was retained for statistical analysis. For sensitivity analysis, the 888, 848 eligible six-IGHV sequence-level records were clustered with MMseqs2 easy-linclust using a minimum CDRH3 amino-acid identity of 0.85 and a minimum alignment coverage of 0.80 (cov-mode 0). Clustering was run within each IGHV gene, and the final 85%-identity CDRH3 cluster identifier additionally retained IGHJ gene and CDRH3 amino-acid length. Donor identity and light-chain information were not used as clustering features. Modal D-gene, light-chain V-gene, and light-chain J-gene calls were retained only after clustering to recalculate V-D, conditional D-J, and VH-VL summaries. This sensitivity analysis tests robustness to a more permissive CDRH3-based collapsing rule but does not reconstruct donor-resolved clonal lineages. Exact-clonotype and 85%-identity CDRH3-cluster results were compared by both enrichment/depletion direction and log2(O/E) effect size.

### V(D)J usage and association analysis

V-D, V-J, and V-conditioned D-J usage were quantified at the exact-clonotype level for the six selected IGHV genes. For marginal V-D and V-J analyses, observed gene-pair counts were compared with expected counts under an independence model, `Eij = (Ri x Cj)/N`, where `Ri` and `Cj` are row and column totals and `N` is the total number of observations. For V-conditioned D-J analyses, each IGHV background was analyzed separately. Within a given IGHV background `v`, expected D-J counts were calculated as `EDJ|v = (Dv x Jv)/Nv`, where `Dv` and `Jv` are the D- and J-gene marginal totals and `Nv` is the number of exact clonotypes in that IGHV background. For the primary OAS V-D and V-conditioned D-J analyses, enrichment was calculated directly as log2(O/E) for cells with positive observed and expected counts. Cells with an expected count of zero were considered not evaluable. A 0.5 pseudocount was used only in the explicitly identified metadata-stratified VH-VL sensitivity analysis. Sankey diagrams were used for descriptive visualization of V(D)J trajectories.

Haberman standardized residuals (60) were calculated for exploratory heatmaps but were not used as stand-alone evidence of biologically meaningful enrichment or depletion because residual magnitude can increase with sample size. Primary interpretation followed an effect-size-first framework based on observed support, expected support, log_2_(O/E), study-level bootstrap confidence intervals, and study-aware concordance. Associations were emphasized when they met predefined criteria: observed count ≥50, expected count ≥10, absolute log_2_(O/E) ≥0.5, and a bootstrap confidence interval supporting the same direction.

Study-level 95% bootstrap confidence intervals were estimated by resampling the 17 validated OAS DS Name/source-study groups with replacement for 1, 000 replicates and recalculating direct log2(O/E) for evaluable cells. To assess robustness to study composition, key V-D and V-conditioned D-J effects were also recalculated within individual studies where sufficient counts were available. Study-robust associations required at least three evaluable studies and ≥70% directional concordance with the pooled result. Donor-level and platform-level mixed-effects models were not applied uniformly because these metadata were incomplete or inconsistently annotated across OAS source files. The study- and metadata-stratified analyses were therefore used to assess robustness of direction rather than to estimate donor-independent or platform-independent effects.

Productive versus non-productive V-D and D-J comparisons were prespecified as conditional analyses if a native non-productive subset was present. Because the source-level audit identified no eligible non-productive records, these comparisons were not estimable and no non-productive group was reconstructed from frameshift, stop-codon, reverse-complement, or missing-value fields.

### Heavy-light chain pairing analysis

Using paired OAS sequence information, we constructed heavy-chain V and light-chain V pairing matrices for kappa and lambda chains. For each VH-VL combination, an odds ratio and two-sided Fisher’s exact-test P value were calculated from a 2 x 2 table contrasting the focal VH-VL cell with the corresponding non-focal VH, non-focal VL, and remaining repertoire counts. P values were adjusted across the 395 tested VH-VL combinations using the Benjamini-Hochberg false discovery rate procedure. For the primary pooled OAS analysis and study-level bootstrap analysis, log2(O/E) was calculated directly for cells with positive observed and expected counts. For consistency with the effect-size-first V(D)J analysis, VH-VL pairings were also evaluated using study-level bootstrap confidence intervals for log2(O/E). Residual heatmaps were retained only as exploratory visualizations, whereas major VH-VL pairing calls required observed and expected count support, an absolute log2(O/E) threshold of 0.5, bootstrap CI direction consistency, and study-level concordance.

Selected key pairings were further evaluated by leave-one-study-out recalculation, per-study log2(O/E) estimation where evaluable, and sensitivity analyses based on the available disease and vaccine metadata. In addition to the broad immune-state grouping, source files were examined within disease/vaccine-defined metadata contexts. Within each study-context stratum, observed and expected counts were recalculated under the independence model, and log2[(Observed + 0.5)/(Expected + 0.5)] was used to summarize the direction and magnitude of the association. The pseudocount-adjusted effect size was used only for this sparse, metadata-stratified sensitivity analysis and was not used for the primary pooled OAS effect-size calls. A cell was considered evaluable when the observed count was at least 5 and the expected count was at least 1; context-level summaries were interpreted cautiously when fewer than three studies were evaluable. These analyses were used to distinguish strong pooled signals from signals that were study- or annotation-sensitive.

### External comparison with PairedAbNGS

To evaluate whether the OAS-derived VH-VL and CDRH3 patterns were reproducible in an external paired-repertoire resource, we analyzed the public PairedAbNGS FASTA archive. FASTA records were parsed to extract BioProject, run accession, species, heavy-chain gene calls, light-chain gene calls, CDRH3, CDRL3, and paired heavy- and light-chain variable-region amino-acid sequences. Gene calls were standardized to gene level by removing allele suffixes. We retained human records with interpretable heavy-chain V/J, light-chain V/J, valid CDRH3 and CDRL3 amino-acid sequences, and one of the six focus IGHV genes. PairedAbNGS records were collapsed to exact paired clonotypes using VH|JH|CDRH3|VL|JL|CDRL3.

Potential OAS-PairedAbNGS overlap was evaluated at source-run, exact paired heavy/light amino-acid sequence, and exact paired-clonotype levels. Because the OAS structured metadata did not provide explicit BioProject identifiers, BioProject-level overlap was not treated as evaluable. VH-VL observed/expected effect sizes were recalculated in PairedAbNGS using the same independence model and direct log2(O/E) definition as in the primary OAS analysis. Effect sizes were calculated only for cells with positive observed and expected counts; cells with absent marginals or zero observed counts were classified as not evaluable. No pseudocount was used in the primary cross-resource comparison. CDRH3 length, simplified side-chain charge score, and Kyte-Doolittle GRAVY hydropathy were summarized by IGHV gene. All analyses were repeated after excluding PairedAbNGS records with identifiable source-run, exact paired-sequence, or exact paired-clonotype overlap with OAS.

As an additional sensitivity analysis, IGHD calls were standardized to gene level, and V-conditioned D-J frequencies and direct log2(O/E) values were recalculated within each IGHV background using the 22 IGHD genes shared with the OAS conditional D-J matrix. Cells with positive observed and expected counts were considered evaluable, and no pseudocount was used. These D-J results were treated as supplementary cross-resource sensitivity analyses rather than as evidence of universal D-J conservation or primary recombination preferences.

### Light-chain coherence sensitivity analysis

To relate the context-stratified pairing results to light-chain coherence, we first summarized VH-conditioned light-chain V-gene concentration within each study and metadata group using the top-VL fraction, Simpson concentration, and effective VL number. We then performed an exploratory shared-heavy analysis in source files with a strict single-donor identifier. Donors were compared only within the same study and metadata group when they carried the same VH gene and identical CDRH3 amino-acid sequence. One modal light-chain V gene was retained per donor and shared-heavy clonotype, ties were excluded, and D-named light-chain V-gene paralogs were collapsed. The observed proportion of cross-donor pairs using the same light-chain V gene was compared with the corresponding within-study, VH-specific marginal expectation. As uniform naive and memory B-cell subset annotations were not available, this analysis was used as a sensitivity analysis related to light-chain coherence rather than as a direct replication of naive-versus-memory coherence estimates.

### Junctional diversity and nucleotide composition

Junctional diversity is a major source of antibody specificity.

We analyzed the nucleotide composition and length distribution of CDR3-coding junctional regions and their flanking sequence features. Specifically, we quantified junction nucleotide composition, GC content, junction length distributions, and the correlation between junction length and GC content. Junctional region nucleotide composition and length distributions were calculated from the source `junction_heavy` field. A separate DH reading-frame analysis was not performed because the merged OAS analysis table did not retain the D-segment alignment coordinates required for reliable frame assignment, such as D-sequence start/end, D-germline start/end, or a native D-frame field. These analyses were used to characterize broad sequence-level constraints in productive antibody rearrangements rather than to infer the exact enzymatic history of individual sequences.

### CDRH3 physicochemical features and sequence liability proxies

To characterize IGHV-associated CDRH3 sequence features, we calculated CDRH3 amino-acid length from the IMGT-style cdr3_aa_heavy field, a simplified side-chain charge score, GRAVY hydropathy, and amino acid enrichment or depletion relative to the global repertoire baseline. For the primary charge summary, Asp (D) and Glu (E) were assigned a value of -1, Lys (K) and Arg (R) were assigned a value of +1, His (H) was assigned a value of +0.1, and other amino acids were assigned a value of 0. The His value was used as a Henderson-Hasselbalch approximation of partial imidazole protonation near physiological pH. To evaluate sensitivity to this convention, we repeated the charge calculation using a pKa-based side-chain model at pH 7.4 with published protein pKa values (61). Because CDRH3 is an internal antibody loop, N- and C-terminal peptide charges were not included in this sensitivity analysis. Hydropathy was calculated using the Kyte-Doolittle GRAVY scale (62). Length distributions were summarized at each integer amino-acid length; the nucleotide junction_length_heavy field was used only for field and frame auditing.

For each IGHV gene, we summarized median values, interquartile ranges, and 10th-90th percentile intervals for CDRH3 length, side-chain charge score, and hydropathy. We also calculated lightweight CDRH3-level sequence liability proxies, including absolute side-chain charge score, GRAVY hydropathy, hydrophobic residue fraction, aromatic residue fraction, and liability motif counts. The liability motif count included potential N-linked glycosylation motifs (N[^P][ST]), deamidation-prone NG and NS motifs, and methionine residues as oxidation-prone sites.

The descriptive sequence-liability proxy was calculated as z(|side-chain charge score|) + z(GRAVY) + z(hydrophobic-residue fraction) + z(liability-motif count), with each component standardized across the pooled six-IGHV exact-clonotype population. This composite was used only as a descriptive sequence-level summary and was not validated against experimental expression, stability or developability outcomes.

### Hypothesis-based analysis of the IGHV4-34-associated CDRH3 side-chain charge-score profile

To test the prespecified IGHV4–34 hypothesis, we compared CDRH3 side-chain charge in IGHV4–34 exact-clonotype representatives with the pooled charge of representatives assigned to the other five focus IGHV genes. The CDRH3-only population contained 712, 148 representatives; the paired-gene analysis population contained 712, 143 because five representatives had valid CDRH3 sequences but no interpretable light-chain V-gene call. Pooled OAS analyses retained one record per global exact clone identifier. Study-level analyses deduplicated clone identifiers within each DS Name, and immune-context analyses deduplicated clone identifiers within each DS Name and context class. A study or study-context unit was considered evaluable when it contained at least 50 IGHV4–34 clonotypes and at least three comparator IGHV backgrounds with at least 50 clonotypes each.

Within each eligible unit, we calculated the difference in mean CDRH3 side-chain charge between IGHV4–34 and the comparator backgrounds. Study-level effects were summarized using a DerSimonian-Laird random-effects model with Hartung-Knapp confidence intervals and leave-one-study-out sensitivity analysis. Sensitivity analyses equally weighted the five comparator IGHV means, adjusted the charge contrast for CDRH3 amino-acid length, calculated charge per residue, and repeated the analysis using a pKa-based side-chain charge model at pH 7.4. Immune-context summaries required at least three evaluable studies for inferential reporting; categories with fewer studies were treated descriptively. The same contrast was recalculated in PairedAbNGS and summarized across BioProjects before and after strict exclusion of clonotypes with identifiable OAS overlap by run accession, paired amino-acid sequence, or exact paired-clonotype key.

### Statistical analysis and visualization

All data processing and statistical analyses were conducted in Python 3.8. The primary libraries included Pandas (v1.3.0) (63) for data cleaning and tabulation, NumPy (v1.21.0) (64) for numerical operations, SciPy (v1.7.0) (65) for statistical tests, Matplotlib (v3.4.0) (66) and Seaborn (v0.11.0) (67) for static visualizations, and Plotly for Sankey diagrams.

## Results

### Selected IGHV backgrounds show recurrent V(D)J usage patterns in expressed paired repertoires

We mapped V(D)J usage around the six focus IGHV genes using 712, 143 exact clonotypes after gene-level standardization and clonotype collapsing (**Supplementary Figure S1**). V(D)J usage was not uniform across the paired repertoire. Each IGHV background showed preferentially represented paths involving selected D and J genes, as illustrated by the V(D)J trajectory summaries and complete Sankey maps (**Figure 2**; **Supplementary Figure S2**), a compact summary of dominant gene usage is provided in **Supplementary Figure S3**.

**Figure 2:**
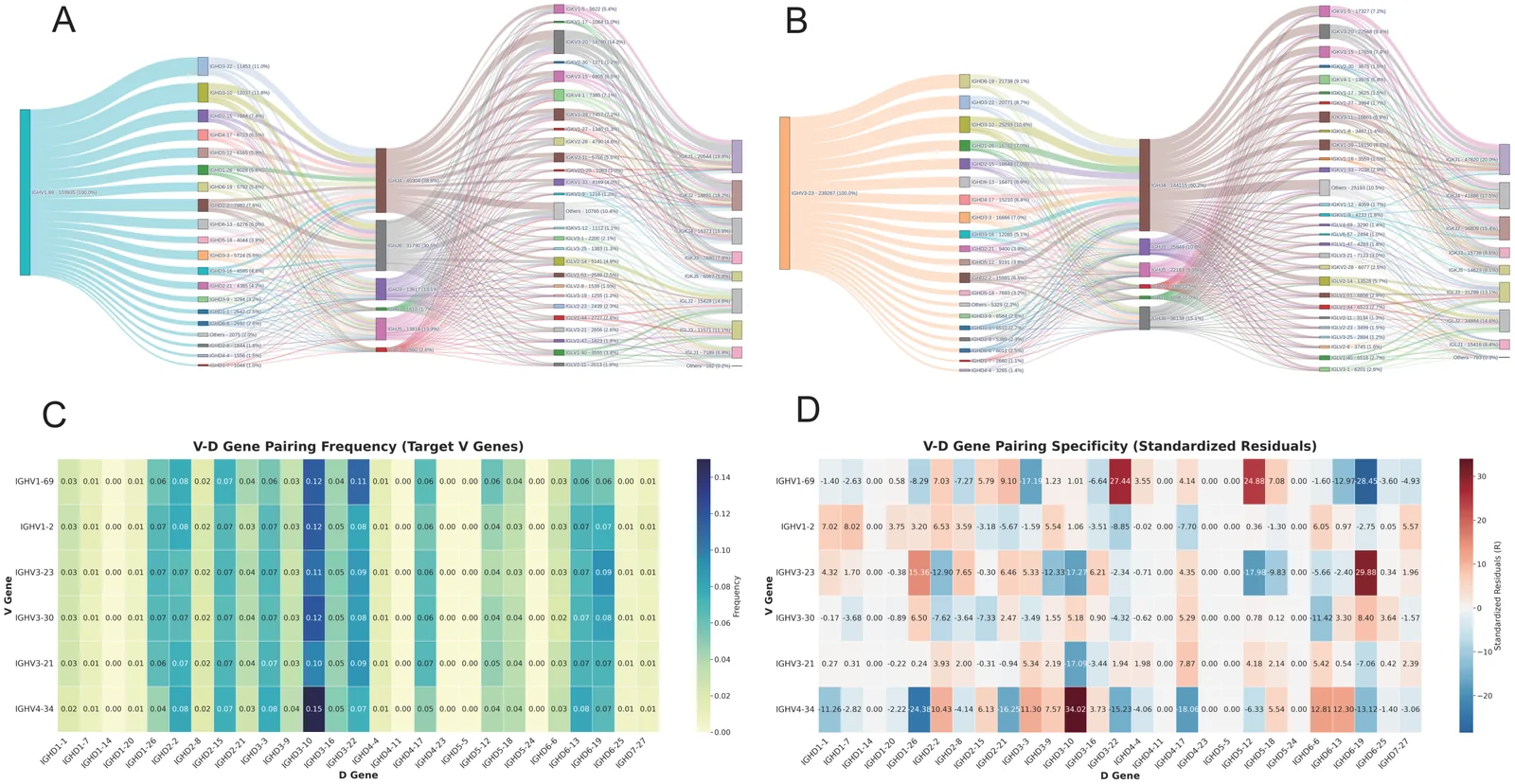
Selected IGHV-associated V(D)J usage patterns in expressed paired repertoires **(A, B)** Representative Sankey diagrams illustrate observed usage trajectories flowing from specific selected V genes (such as IGHV1–69 and IGHV3-23) to D and J gene segments; line thickness reflects relative path abundance. **(C)** Global V-D gene pairing frequency matrix for six selected heavy-chain V genes. **(D)** Standardized residuals of V-D pairing frequency, retained as exploratory visualization of deviations from expectation under the independence model. Positive values indicate enrichment relative to marginal expectation, whereas negative values indicate depletion.

At the V-D level, several D genes were frequently used across multiple V-gene backgrounds. IGHD3–10 was among the most common D genes, with usage frequencies of approximately 0.10-0.15 in several IGHV groups. Because high frequency can reflect basal gene abundance, annotation frequency, or study composition, we interpreted these descriptive patterns together with effect-size and study-aware analyses. Marginal V-D effects were generally modest. Only one of 132 associations met all prespecified count-support, effect-size, bootstrap-CI, and study-concordance criteria: IGHV4-34-IGHD1–26 was depleted relative to expectation [log2(O/E) = -0.519]. IGHV1-69-IGHD5–12 [log2(O/E) = 0.412] and IGHV1-69-IGHD6–19 [log2(O/E) = -0.477] showed high study-level directional concordance but did not meet the prespecified absolute effect-size threshold and were therefore not classified as major V-D effects (**Supplementary Table 2**).

We next examined whether conditional D-J usage differed across the six selected IGHV backgrounds. Conditional D-J frequency heatmaps showed recurrent use of IGHJ4 across the six IGHV backgrounds (**Supplementary Figure S4**), indicating a shared J-gene usage component. After adjustment for marginal D- and J-gene usage, the conditional D-J landscape showed stronger and more recurrent signals than the marginal V-D analysis (**Supplementary Figure S5**; **Supplementary Table 2**). For example, IGHD2–2 was consistently enriched with IGHJ6 and depleted with IGHJ4 across several V backgrounds, and IGHD3–3 showed a similar IGHJ6-enriched and IGHJ4-depleted pattern. IGHD3-10, despite its high overall usage, shifted between relative enrichment and depletion depending on the V background. These results indicate that D-J usage contains recurrent modules, while the magnitude and direction of specific D-J associations remain associated with IGHV background.

We also assessed whether non-productive rearrangements were available to distinguish rearrangement-associated from selection-associated usage. Native-schema auditing of 580 paired OAS source files covering 3, 003, 127 records identified no heavy- or light-chain records annotated as non-productive. Therefore, productive versus non-productive V-D and D-J comparisons could not be estimated from the paired source release. These observations should therefore be interpreted as patterns in the productive paired OAS release analyzed here, rather than as direct estimates of primary rearrangement preferences (**Supplementary Table 3**).

To place the six focus genes in the broader IGHV repertoire, we also summarized all sufficiently abundant IGHV genes. Among 2, 965, 731 records with valid standardized IGHV assignment, 53 IGHV genes were detected, and 22 genes had at least 50, 000 sequence-level records. These high-abundance genes accounted for 80.81% of valid IGHV-assigned records, and all six focus genes passed this threshold. Thus, the six genes should not be viewed as an unbiased IGHV-wide screen; rather, they represent a biologically motivated, high-support subset within the broader expressed repertoire landscape (**Supplementary Table 4**).

### Effect-size and sensitivity analyses identify recurrent VH-VL pairing patterns

To describe the sampling context of the paired dataset, we summarized the available disease and vaccine metadata. Among 2, 965, 738 source-level paired-record counts represented in the structured metadata, 2, 388, 353 (80.53%) had an explicit disease and/or vaccine annotation. Infectious-disease and vaccinated/convalescent sources accounted for the largest fractions, whereas 577, 385 records (19.47%) were unclassified or unannotated. Because the metadata did not uniformly identify naive and memory B-cell subsets, the unclassified/unannotated group was not used as a healthy or naive reference (**Supplementary Figure 6**; **Supplementary Table 5**).

We assessed whether paired OAS repertoires contained IGHV-associated VH-VL patterns and whether the strongest signals persisted across available metadata groups. IGKV3–20 and IGKV1–39 were frequent across several heavy-chain backgrounds (**Figure 3A**; **Supplementary Figure S7**), but high marginal usage is not evidence of enrichment. Residual heatmaps were therefore exploratory; primary interpretation used observed and expected counts, log2(O/E), FDR-adjusted statistics, study-level bootstrap confidence intervals, and study concordance (**Figures 3B, C**; **Supplementary Tables 2**, **6**). The effect-size-first analysis identified recurrent IGHV-associated VH-VL enrichment and depletion patterns. Among 395 associations, 81 met the predefined count-support, absolute log2(O/E), bootstrap-direction, and study-concordance criteria, including IGHV4-34-IGLV3-19, IGHV1-69-IGKV3-20, and IGHV4-34-IGKV1-39; associations lacking sufficient support or bootstrap direction were not treated as major pairing calls (**Supplementary Table 2**; **Supplementary Figure S8**). All 11 key VH-VL pairs retained their direction in leave-one-study-out analyses, and 9 of 11 retained the pooled direction across six broad immune-state groups. Across disease/vaccine-defined contexts, nine pairs were concordant in every evaluable context, whereas IGHV3-30-IGLV3–21 and IGHV1-69-IGKV3–11 were less stable, showing concordance in 7 of 8 and 5 of 8 contexts, respectively. These results indicate that the strongest VH-VL associations recur across OAS study groups and metadata-defined immune contexts, whereas weaker associations remain sensitive to metadata composition (**Figure 4**; **Supplementary Figure 9**; **Supplementary Table 5**).

**Figure 3:**
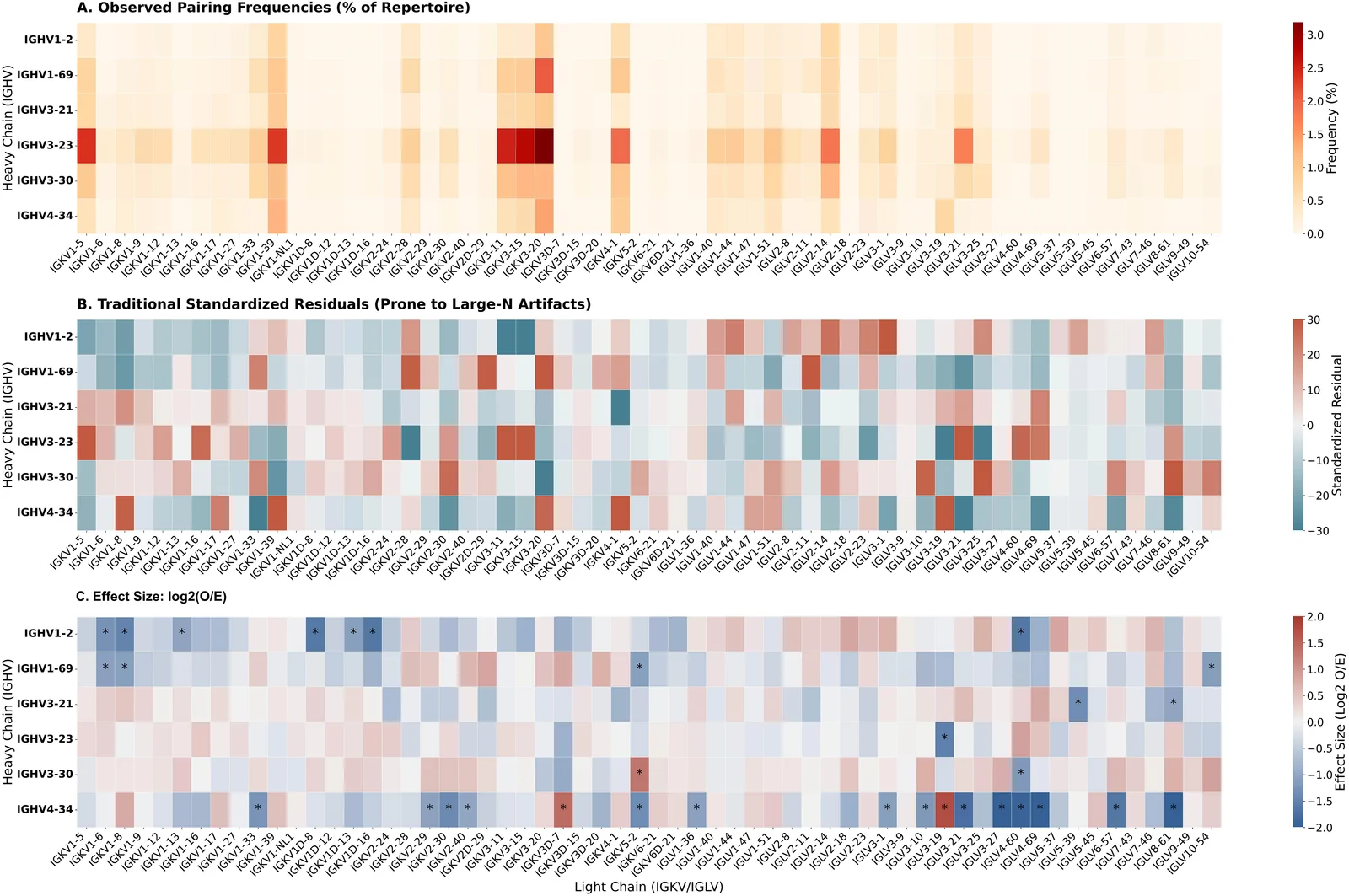
VH-VL pairing patterns in the human paired antibody repertoire. **(A)** Observed VH-VL pairing frequencies across the six selected IGHV genes. **(B)** Standardized residuals under the independence model, shown as an exploratory view of enrichment and depletion. **(C)** Effect-size heatmap showing direct log_2_ observed/expected [log_2_(O/E)] values for VH-VL pairings with positive observed and expected counts. An asterisk denotes a VH-VL association with an FDR-adjusted P value < 0.05, as reported in **Supplementary Table S6**.

**Figure 4:**
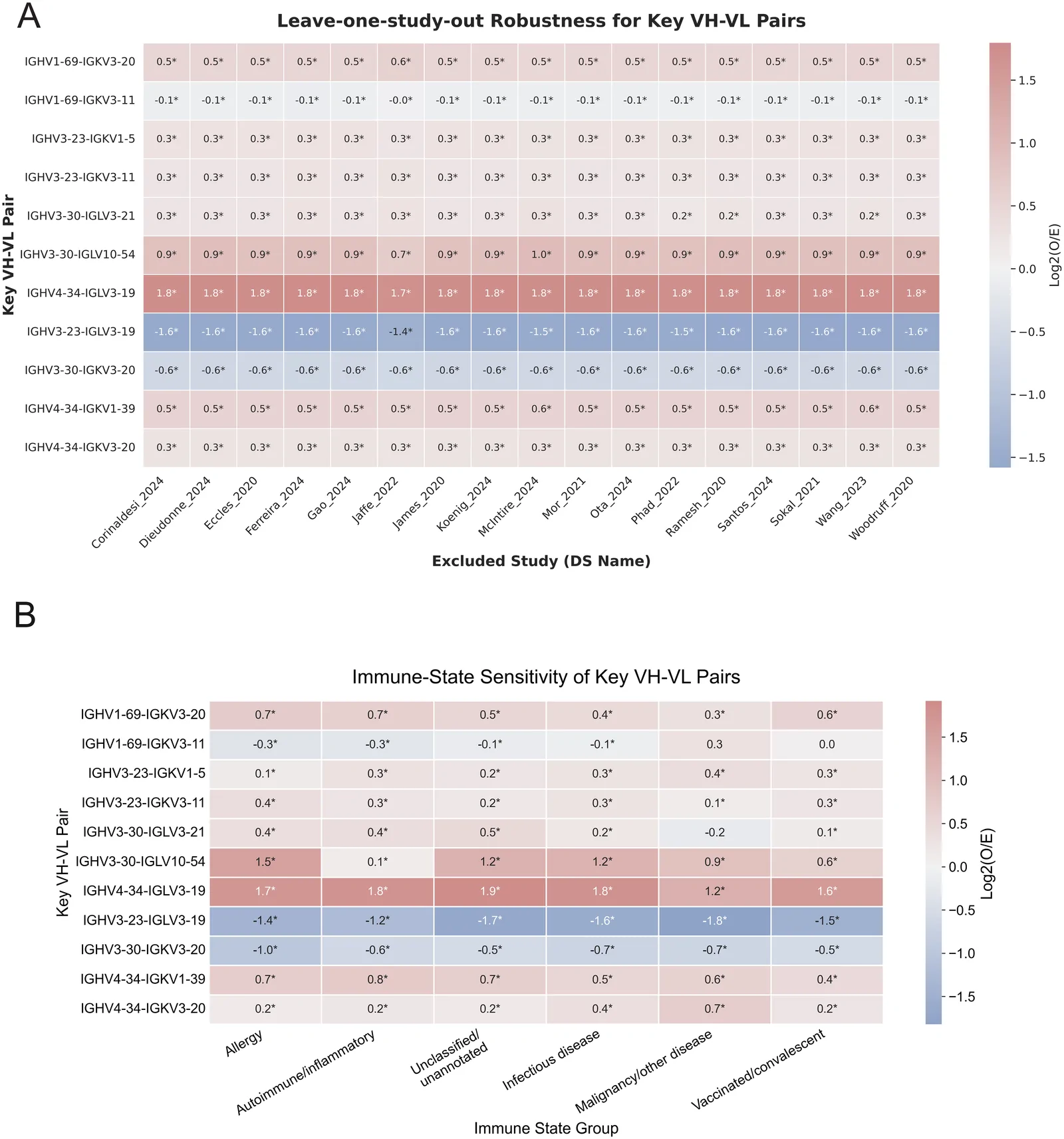
Robustness analyses for key VH-VL pairing patterns. **(A)** Leave-one-study-out direct log2(O/E) values for 11 prespecified key VH-VL pairings after excluding one source-study group at a time. **(B)** Metadata-defined immune-context sensitivity analysis for the same pairings. Asterisks denote evaluable estimates that retained the pooled direction. An asterisk denotes an evaluable leave-one-study-out or immune-context estimate with positive observed and expected counts that retained the direction of the pooled analysis.

We next examined whether the context-stratified VH-VL patterns were compatible with light-chain coherence, a phenomenon previously reported to be more pronounced in functional antibodies than in naive repertoires (73). VH-conditioned light-chain concentration was not consistently higher in disease- or vaccine-annotated studies: the median study-VH Simpson concentration was 0.0492 in annotated contexts and 0.0608 in unclassified or unannotated contexts. In a donor-restricted analysis of recurrent exact shared-heavy clonotypes, 1, 592 of 1, 763 cross-donor pairs used the same light-chain V gene (90.30%; Wilson 95% CI, 88.83%-91.60%), compared with a weighted within-study VH-marginal expectation of 4.59%. This estimate remained similar after exclusion of the study contributing the Jaffe et al. dataset (92.66%, 1, 515/1, 635 pairs; baseline, 4.58%). However, only three studies contributed at least 50 shared-heavy pairs, and uniform naive or memory B-cell annotations were unavailable. We therefore interpret this result as an exploratory light-chain coherence proxy that is compatible with, but does not directly reproduce, the functional-versus-naive comparison reported by Jaffe et al. (73) (**Supplementary Figure S10**; **Supplementary Table 5**). Taken together, these analyses are compatible with a contribution from selection-associated light-chain coherence and argue against interpreting the pooled VH-VL associations as germline-level pairing preferences alone.

To test sensitivity to a more permissive CDRH3-based collapsing rule, we re-clustered the six-IGHV sequence-level records into 85%-identity CDRH3 clusters. This reduced 888, 848 sequence-level records to 656, 052 clusters, corresponding to a 26.19% reduction from the sequence-level input and a 7.88% reduction relative to the exact-clonotype count. The enrichment or depletion direction was preserved for all 11 key VH-VL pairs, all 40 evaluated V-D effects, and all 120 evaluated V-conditioned D-J effects. Effect sizes were also highly concordant between exact clonotypes and 85%-identity CDRH3 clusters: median absolute delta log2(O/E) values were 0.0089 for VH-VL, 0.0108 for V-D, and 0.0261 for V-conditioned D-J comparisons, with corresponding Pearson correlations of 0.9998, 0.9973, and 0.9967. These results indicate that the major association patterns were not dependent on the exact-clonotype boundary, while the 85%-identity analysis should not be interpreted as donor-resolved lineage reconstruction (**Supplementary Table 7**).

Light-chain V-J usage was comparatively stable across heavy-chain backgrounds (**Supplementary Figure S11**). IGKV genes predominantly used IGKJ1 and IGKJ2, whereas IGLV genes mainly used IGLJ2 and IGLJ3. Thus, the strongest IGHV-linked variation was observed in inter-chain VH-VL associations rather than in these broad intra-chain V-J patterns.

### Sequence features of productive CDRH3-coding junctions

CDRH3 makes a major contribution to antigen-contact diversity. We therefore examined the sequence and physicochemical distributions of CDRH3 in productive expressed human antibodies and report them as empirical reference ranges.

Junction lengths were summarized at integer amino-acid lengths and not used to infer DH reading-frame usage. At the sequence level, productive CDRH3-coding junctional regions showed recurrent nucleotide and length distributions rather than uniform composition (**Figure 5**). Because these analyses were performed on productive expressed antibodies, they should be interpreted as empirical sequence features of retained repertoires rather than direct evidence of DH-frame selection, V(D)J recombination mechanisms, or specific structural interactions. At the nucleotide level, junctional regions showed enrichment of guanine (G) and thymine (T) (**Figure 5A**), with GC-content distributions centered above 50% across V-gene backgrounds (**Figure 5B**). Junction length distributions centered near 50 bp (**Figure 5C**), and junction length was modestly inversely associated with GC content (**Figure 5D**).

**Figure 5:**
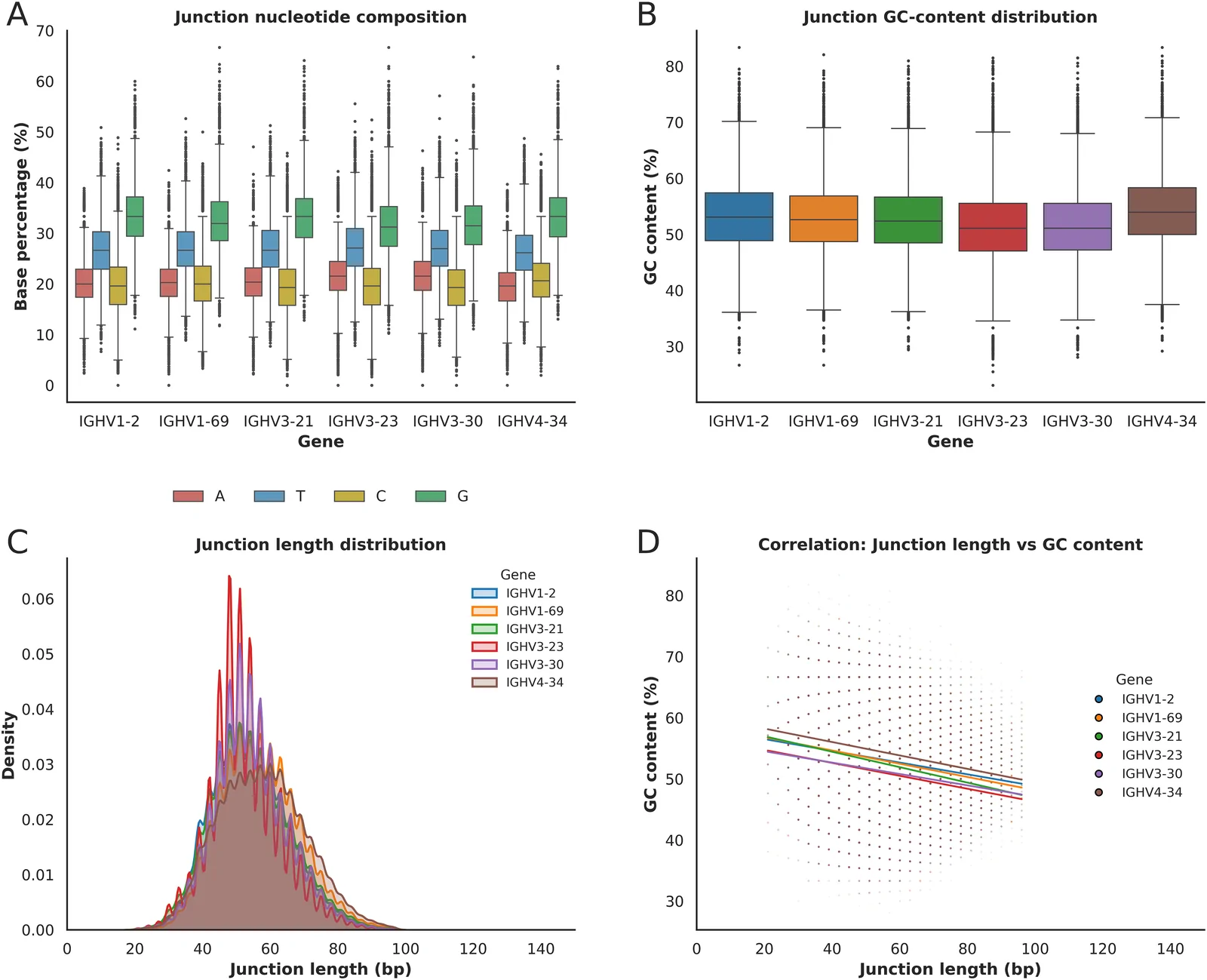
Productive CDRH3-coding junctional sequence features in expressed human antibodies. The junctional region refers to the nucleotide “junction_heavy” field from the source annotation files. **(A)** Junction nucleotide composition. **(B)** Junction GC-content distribution. **(C)** Junction length distribution. **(D)** Correlation between junction length and GC content.

These nucleotide-level summaries define empirical distributions for productive expressed CDRH3 sequences. They can inform repertoire-matched library design without imposing exclusion rules on sequences outside the observed ranges.

### Selected IGHV backgrounds are associated with CDRH3 physicochemical profiles and sequence-liability patterns

We next examined whether CDRH3 physicochemical profiles differed across the six IGHV backgrounds. The analysis focused on side-chain charge score, hydropathy, length, and composition-based liability proxies (**Figure 6**; **Supplementary Figure S12**; **Supplementary Table 8**, **9**). Side-chain charge-score distributions were centered near neutral to slightly negative values for most IGHV-associated CDRH3 repertoires, whereas IGHV4–34 showed a positive-shifted profile with a median score of 0.2 and an interquartile range of 0.0 to 2.0 (**Figure 6A**; **Supplementary Table 8**; charge-calculation sensitivity is summarized in **Supplementary Table 10**). Hydropathy distributions were consistently shifted toward negative Kyte-Doolittle GRAVY values across all six IGHV genes, supporting a broadly hydrophilic CDRH3 sequence space (**Figure 6B**).

**Figure 6:**
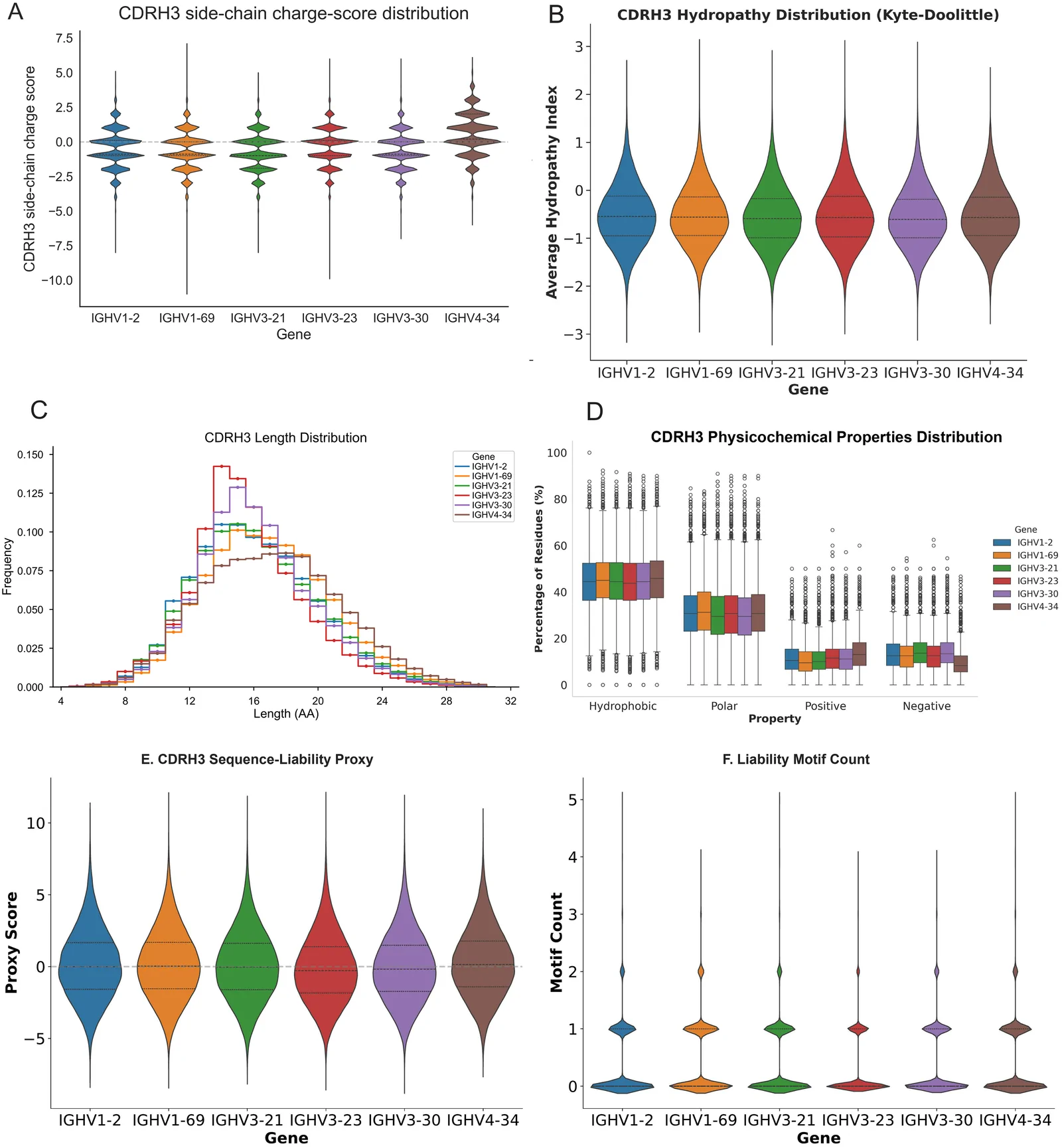
IGHV-associated CDRH3 physicochemical distributions and sequence-liability proxies in expressed paired repertoires. **(A)** Distribution of the CDRH3 side-chain charge score across the six core IGHV backgrounds, calculated as Asp/Glu = -1, Lys/Arg = +1, His = +0.1, and all other residues = 0. **(B)** CDRH3 hydropathy distribution based on the Kyte-Doolittle GRAVY scale. **(C)** Discrete empirical CDRH3 amino-acid length distributions across the six selected IGHV backgrounds. Step outlines show exact-clonotype proportions in one-amino-acid bins, and points mark observed integer lengths. No kernel-density estimation or continuous smoothing was applied. **(D)** Distribution of broad CDRH3 physicochemical residue-class fractions, including hydrophobic, polar, positively charged, and negatively charged residues. **(E)** Distribution of the descriptive CDRH3 sequence-based proxy, calculated as the sum of standardized absolute side-chain charge score, GRAVY, hydrophobic-residue fraction, and liability-motif count. **(F)** Distribution of liability motif counts, including potential N-linked glycosylation, deamidation-prone, and oxidation-prone motifs.

Discrete empirical length analysis showed that individual V genes were associated with distinct CDRH3 amino-acid length distributions (**Figure 6C**). Independent field auditing and count recalculation confirmed that the distribution was derived from cdr3_aa_heavy, rather than nucleotide junction length, and reproduced all 156 IGHV-by-length source-table cells (**Supplementary Table 11**). The median CDRH3 length ranged from 15 amino acids for IGHV3–23 to 17 amino acids for IGHV1–69 and IGHV4-34, with the corresponding empirical 10th-90th percentile intervals provided in **Supplementary Table 8**. Broad residue-class analysis showed comparable hydrophobic and polar residue fractions across the six IGHV backgrounds, with more restricted distributions for positively and negatively charged residue fractions (**Figure 6D**). We additionally calculated a descriptive CDRH3 sequence-based proxy and liability motif counts from CDRH3 composition (**Figures 6E, F**; **Supplementary Table 9**). Median proxy scores were close to zero across the six IGHV genes, with IGHV4–34 showing the highest median proxy score (0.136) and IGHV3–23 showing the lowest median proxy score (-0.286). Liability motif counts were low overall, with mean counts ranging from 0.31 for IGHV3–23 to 0.47 for IGHV1-69. These findings outline IGHV-associated CDRH3 physicochemical ranges and composition-based proxy indicators that can be used as empirical reference ranges for repertoire comparison; the IGHV4-34-associated side-chain charge-score shift was evaluated separately as a study-aware, hypothesis-generating phenotype. A compact supplementary visualization of CDRH3 terminal-position amino-acid profiles is provided in **Supplementary Figure S13**.

### Cross-resource comparison of VH-VL, CDRH3, and D-J patterns with PairedAbNGS

We next compared the OAS-derived key VH-VL and CDRH3 findings against PairedAbNGS. The published PairedAbNGS resource contains more than 14 million paired sequences from 58 bioprojects. The supplied archive contained 58 FASTA members, including 44 human members. After parsing and quality control, 6, 542, 393 valid human paired records were retained, including 1, 944, 381 six-IGHV sequence-level records and 753, 992 six-IGHV exact paired clonotypes. The lower retained count relative to the full PairedAbNGS collection reflects restriction to human records and the requirement for interpretable paired heavy- and light-chain annotations and sequence fields needed for the present analyses. Identifiable overlap with OAS was limited but non-negligible: 47 of 2, 101 PairedAbNGS run accessions overlapped with OAS source-run accessions (2.24%), 142, 703 six-IGHV sequence-level rows matched an OAS paired heavy/light amino-acid sequence hash (7.34%), and 53, 568 exact paired clonotypes overlapped with OAS (7.10%). After excluding records with identifiable run-accession, exact paired-sequence, or exact paired-clonotype overlap, 631, 976 six-IGHV exact paired clonotypes remained.

Among the 11 prespecified OAS key VH-VL pairs, 10 were evaluable in PairedAbNGS; IGHV4-34-IGKV1–39 was not evaluable because IGKV1–39 had no marginal support in the parsed PairedAbNGS six-IGHV matrix. Eight of the 10 evaluable pairs retained the same enrichment or depletion direction, and the OAS and PairedAbNGS direct log2(O/E) values were positively correlated (Pearson r = 0.888; Spearman r_s = 0.673; **Figure 7**; **Supplementary Table 12**). After identifiable-overlap exclusion, all 10 evaluable pairs preserved their PairedAbNGS direction relative to the all-record analysis, whereas concordance with OAS remained 8 of 10 (Pearson r = 0.851; Spearman r_s = 0.600; **Supplementary Figure S14**). IGHV1-69-IGKV3–11 and IGHV3-23-IGKV3–11 showed discordant directions.

**Figure 7:**
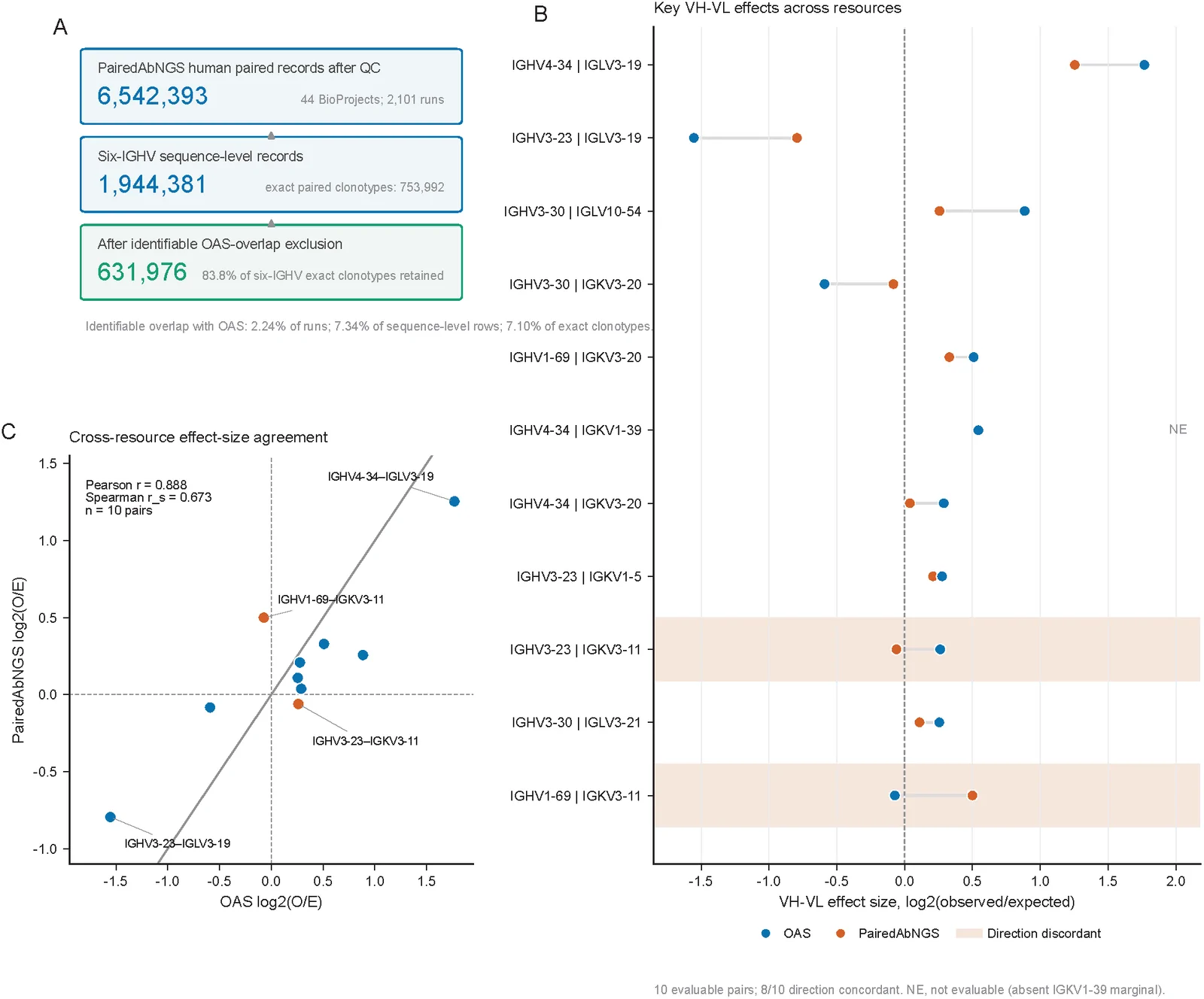
Cross-resource evaluation of key VH-VL associations in PairedAbNGS. **(A)** Records retained after parsing and quality control, restriction to the six focus IGHV genes, exact paired-clonotype collapsing, and exclusion of records with identifiable OAS overlap. **(B)** OAS and PairedAbNGS direct log2(O/E) values for the 11 prespecified key VH-VL pairs. Effect sizes were calculated as log2(Observed/Expected) only for pairs with positive observed and expected counts; pairs with absent marginals were classified as not evaluable. **(C)** Cross-resource agreement for the 10 evaluable pairs.

PairedAbNGS also provided broad cross-resource support for the IGHV-associated CDRH3 profiles. Across the six IGHV genes, median CDRH3 length, simplified side-chain charge score, and GRAVY hydropathy showed positive descriptive rank correlations between resources (Spearman r_s = 0.876, 0.861, and 0.841, respectively (**Supplementary Figure S15**; **Supplementary Table 12**). IGHV4–34 remained the highest-scoring IGHV background in both resources, and median GRAVY values were negative for all six IGHV genes. These correlations are descriptive because they are based on six IGHV-level observations.

Among PairedAbNGS-supported, OAS study-robust V-conditioned D-J calls, 88 of 91 retained their direction (96.7%); after identifiable-overlap exclusion, 84 of 89 remained concordant (94.4%; **Supplementary Figures S16–18**; **Supplementary Table 13**). Direct log2(O/E) values were used in both resources. Weaker cells were more resource-sensitive. The cross-resource analyses therefore support selected VH-VL, CDRH3, and D-J patterns without establishing universal germline-level rules.

### The IGHV4-34-associated positive CDRH3 side-chain charge-score shift is study-robust and supported across resources

We next tested whether the positive CDRH3 side-chain charge-score shift associated with IGHV4–34 represented a study-robust repertoire association. In pooled OAS exact clonotypes, the mean CDRH3 score was 0.551 for IGHV4–34 and -0.551 for the other five IGHV backgrounds, corresponding to a mean difference of 1.101. A positive score was observed in 55.38% of IGHV4–34 CDRH3 sequences compared with 25.65% of comparator sequences. Fourteen of 17 OAS studies met the predefined support criteria, and all 14 retained a positive mean-score contrast. The random-effects estimate was 1.041 (Hartung-Knapp 95% CI, 0.968-1.115). Although the direction was consistent, the magnitude varied substantially across studies (I2 = 94.66%, **Supplementary Figure S19**), and we therefore interpret this result as directionally robust but quantitatively heterogeneous. The direction remained positive after exclusion of each study in turn and after equal weighting of the five comparator IGHV backgrounds.

The association was not explained by the longer CDRH3 distribution of IGHV4-34. The length-adjusted study-level coefficient was 1.060 (Hartung-Knapp 95% CI, 0.984-1.135), and the length-adjusted pKa-based estimate was 1.065 (Hartung-Knapp 95% CI, 0.989-1.141). The side-chain charge score per residue also remained positive, with a study-level mean difference of 0.0630 (95% CI, 0.0599-0.0658). Infectious-disease and unclassified or unannotated strata contained at least three eligible studies and retained positive study-level summaries. The autoimmune/inflammatory category included only two eligible studies; the exploratory autoimmune-minus-nonautoimmune contrast was 0.035, providing no evidence for autoimmune-context-specific amplification. Unclassified or unannotated data were not interpreted as a healthy naive repertoire. Immune-context estimates are shown in **Supplementary Figure S20**.

PairedAbNGS provided cross-resource support for the IGHV4-34-associated shift. The pooled mean-charge difference was 0.972, and all 38 eligible BioProjects retained a positive direction (random-effects estimate, 0.985; Hartung-Knapp 95% CI, 0.878-1.092). After strict exclusion of 123, 802 exact clonotypes with identifiable OAS overlap from the 753, 992 six-IGHV exact clonotypes in the PairedAbNGS IGHV4–34 analysis set, the pooled difference remained 0.956 and 630, 190 exact clonotypes remained (random-effects estimate, 0.967; Hartung-Knapp 95% CI, 0.851-1.082). Residue-level decomposition indicated that the shift was associated primarily with a higher Arg fraction and lower Asp/Glu fractions rather than enrichment of all positively charged residues (**Supplementary Table 14**). A consolidated summary of the recurrent VH-VL and CDRH3 features across the six selected IGHV genes is provided in **Table 1**.

**Table 1:** Summary of recurrent repertoire features for the six selected IGHV genes.

IGHV gene	Recurrently enriched or depleted VH-VL pairs	CDRH3 empirical 10th-90th percentile interval	Side-chain charge score and GRAVY IQRs	Descriptive sequence-based proxy summary	Interpretation
IGHV1-69	Enriched: IGKV3-20; caution: IGKV1–8 and weakly negative IGKV3-11	12–22 aa	Score -1.0 to 0.0; GRAVY -0.94 to -0.14	Median 0.026; liability motifs mean 0.47	Recurrent IGKV3–20 enrichment; lower-ranked kappa pairs require cautious interpretation.
IGHV1-2	Enriched: IGLV3-1, IGLV2-14, IGLV3-25; depleted: IGKV1-8, IGKV1-6	11–21 aa	Score -1.0 to 0.1; GRAVY -0.95 to -0.12	Median -0.010; liability motifs mean 0.38	Several lambda-supported signals; depleted IGKV1 partners are less represented than expected.
IGHV3-21	Enriched: IGLV3-21, IGLV4-69, IGKV1-39	11–21 aa	Score -1.9 to 0.0; GRAVY -0.99 to -0.17	Median -0.040; liability motifs mean 0.41	Broad empirical CDRH3 length distribution with a modest negative shift in side-chain charge score.
IGHV3-23	Enriched: IGKV1-5, IGKV3-11, IGKV3-15; depleted: IGLV3-19	12–20 aa	Score -1.0 to 0.1; GRAVY -0.97 to -0.13	Median -0.286; liability motifs mean 0.31	Compact CDRH3 profile with relatively low sequence-liability proxy in this dataset.
IGHV3-30	Enriched: IGLV8-61, IGLV6-57, IGLV3-10, IGLV10-54; depleted: IGKV3-20	12–21 aa	Score -1.0 to 0.0; GRAVY -0.99 to -0.19	Median -0.180; liability motifs mean 0.39	Lambda-biased enrichment signals; IGKV3–20 abundance should not be interpreted as compatibility alone.
IGHV4-34	Enriched: IGLV3-19, IGKV1-39, IGKV3-20; depleted: IGLV3-21, IGLV6-57	12–23 aa	Score 0.0 to 2.0; GRAVY -0.95 to -0.14	Median 0.136; liability motifs mean 0.42	Strong recurrent pairing signals with a positively shifted CDRH3 side-chain charge-score profile.

VH-VL entries summarize repertoire-level enrichment or depletion supported by observed counts and log2(O/E), with bootstrap and study-level support where available. CDRH3 intervals summarize empirical distributions in productive expressed repertoires. These observations do not establish structural compatibility, expression, aggregation, stability, or experimental developability.

## Discussion

We mapped repertoire organization around six well-represented, immunologically relevant IGHV genes in a large public paired-antibody dataset. The genes were selected *a priori* for biological relevance and data support, not as an unbiased screen of the IGHV locus. All six were among the 22 IGHV genes represented by at least 50, 000 standardized records, which together accounted for 80.81% of valid IGHV-assigned records.

V(D)J recombination is influenced by recombination signal sequence efficiency, chromatin accessibility, physical organization of antigen-receptor loci, exonuclease trimming, and junctional insertion processes (39–41, 55). Preferential V-D and D-J trajectories observed in expressed repertoires are therefore composite outcomes of recombination accessibility, productive selection, clonal filtering, and dataset composition. The conditional D-J results are nevertheless informative because they show that high basal usage is not the same as a uniform D-J association pattern. D-J modules recurred across IGHV backgrounds, but individual effect sizes and directions varied by background. These conditional D-J patterns indicate V-associated differences in expressed repertoires, but they do not establish a direct causal effect of the upstream V gene on D-J recombination. The paired OAS collection was enriched for disease- and vaccine-annotated samples and did not provide a uniformly identifiable normal-naive comparator. Recurrence across studies and metadata groups therefore argues against a single-cohort explanation for several strong associations, but cannot separate germline availability, productive selection, antigen exposure, clonal expansion, and sampling. No native non-productive comparator was available, so the V-D and D-J results cannot be interpreted as primary rearrangement probabilities. Similarly, because reliable DH reading-frame assignment requires D-segment alignment coordinates that were not retained in the merged analysis table, the junctional and CDRH3 analyses should be interpreted as IGHV-associated sequence-level associations in productive expressed repertoires, not as evidence for DH-frame selection or V(D)J recombination mechanisms. Re-clustering the six-IGHV sequence-level records into 85%-identity CDRH3 clusters preserved every evaluated effect direction and produced highly concordant log2(O/E) effect sizes, indicating that the main associations were not specific to the exact-clonotype boundary. This analysis was not donor-aware and does not reconstruct donor-resolved clonal lineages.

Heavy-light chain pairing is a key layer of paired repertoire biology. Previous studies have shown that endogenous VH-VL pairs can be recovered at scale and that natural pairing is non-random (47). Structural and computational analyses further indicate that VH/VL packing can shape antigen-binding geometry and biophysical behavior (48, 49), and germline-level studies have linked pairing preferences to charge distributions and interface residues (53). Recent work has extended these observations through PairedAbNGS, which identified biased human heavy-light V-gene pairing and conserved inter-chain contacts (54), and ImmunoMatch, which predicts cognate heavy-light pairing from sequence features (55). Our study builds on this literature by asking how VH-VL preferences around six immunologically important IGHV genes relate to V-D-J usage, robustness across OAS studies, immune-state sensitivity, and CDRH3 sequence features.

The enriched VH-VL combinations are repertoire-level associations in productive antibodies. Several strong directions recurred across OAS study groups and disease/vaccine metadata groups, whereas IGHV1-69-IGKV3–11 and IGHV3-30-IGLV3–21 were more context-sensitive. The shared-heavy analysis was compatible with light-chain coherence, but could not distinguish naive from memory compartments. Effect sizes, bootstrap intervals, leave-one-study-out stability, and context concordance reduce dependence on a single study; they do not establish donor-independent recurrence or remove donor-, subset-, isotype-, or platform-level confounding. Future donor-resolved paired BCR sequencing of well-defined naive and antigen-experienced human B-cell populations will be needed to more directly distinguish repertoire-generation biases from post-rearrangement selection-associated light-chain coherence.

The PairedAbNGS comparison provides an external-resource check on the OAS-derived observations. Several of the strongest VH-VL effects, broad IGHV-associated CDRH3 empirical ranges, and study-robust V-conditioned D-J effects received cross-resource support after excluding identifiable overlapping records, arguing against a purely OAS-specific artifact. However, PairedAbNGS did not support all key VH-VL directions, one pair was not evaluable because of absent PairedAbNGS marginal support, and both resources aggregate public repertoire studies with identifiable overlap. These results therefore support selected expressed-repertoire associations across resources rather than universal or causal germline-level pairing rules.

The CDRH3 analyses define sequence and physicochemical ranges among productive expressed antibodies. Previous structural and composition studies link CDR-H3 residues and loop length to conformational and antigen-recognition properties (56, 57), but the present distributions do not identify stabilizing interactions in individual antibodies without structural or experimental validation.

The IGHV-associated empirical CDRH3 ranges provided compact summaries of these repertoire observations. Different IGHV backgrounds were associated with distinct CDRH3 length, side-chain charge score, hydropathy, and amino acid-composition profiles. For example, IGHV3–23 showed a shorter median CDRH3 length and a narrower empirical range, whereas IGHV1–69 and IGHV4–34 supported broader or longer distributions. IGHV4–34 also showed a positively shifted score window and distinctive amino acid preferences. These observations fit the broader idea that framework context and CDRH3 composition jointly shape expressed antibody sequence space, but they should be used as empirical repertoire windows rather than strict structural rules.

The study-aware and cross-resource analyses identify the positive CDRH3 side-chain charge-score shift as a cross-resource-supported IGHV4-34-associated repertoire association rather than a feature driven solely by one OAS study, CDRH3 length or the simplified histidine-charge convention. This observation is compatible with the known tolerance-regulated biology of IGHV4–34 and provides a specific hypothesis for antigen-resolved functional testing. However, the similar direction across the supported immune-context strata, together with the limited number of evaluable autoimmune studies, does not support autoimmune-context-specific amplification. Because the public datasets lack uniform antigen specificity, functional autoreactivity measurements, and naive/memory annotations, the present analysis does not establish autoreactive binding or a causal mechanism. For antibody discovery, these results are best used to generate candidates for testing. Neither recurrent VH-VL usage nor the descriptive CDRH3 sequence-liability proxy establishes expression, stability, aggregation, polyspecificity, or therapeutic suitability.

Repertoire-derived features could also complement sequence-based design models. Recent deep-learning models, such as AntiFormer, illustrate how antibody sequence representations can be integrated with graph-based information for antibody-antigen affinity prediction (68). The IGHV-conditioned CDRH3 profiles and study-robust VH-VL effect sizes reported here could provide repertoire-derived annotations for candidate prioritization, library design, or model-based filtering. However, these features were not trained or evaluated against binding affinity, expression, or developability outcomes. They should therefore be viewed as empirical repertoire context that may complement validated predictive models, rather than as predictors in their own right.

The rationale for selecting these IGHV genes is supported by prior clinical and structural antibody literature. Recurrent genetic lesions can refine prognostic stratification in chronic lymphocytic leukemia (69), while antibodies targeting conserved influenza hemagglutinin epitopes illustrate how recurrent human antibody solutions can arise against structurally constrained viral sites (70, 71). VH4-34-encoded autoreactivity and its regulation during human B-cell tolerance provide relevant background for interpreting IGHV4–34 as an immunologically distinctive germline context (72). The cross-study and cross-resource charge analysis is compatible with this tolerance-regulated biology and supports a testable repertoire-level hypothesis, but it does not establish a disease mechanism or direct autoreactive binding from pooled public repertoire data.

Overall, this analysis links V-associated conditional D-J usage patterns, VH-VL associations, and CDRH3 sequence profiles within six defined IGHV backgrounds. Study-aware sensitivity analyses and PairedAbNGS comparisons support several of the strongest patterns, including the positive IGHV4-34-associated CDRH3 side-chain charge-score shift. The findings remain expressed-repertoire associations rather than universal rules for all IGHV genes or donors. They provide a reference for condition-matched validation and for selecting candidate heavy-light combinations for subsequent functional or structural evaluation.

## Data Availability

All antibody repertoire records analyzed in this study were obtained from publicly available resources. The primary paired heavy- and light-chain repertoire data were retrieved from the Observed Antibody Space (OAS) database (https://opig.stats.ox.ac.uk/webapps/oas/). **Supplementary Table 1** provides a structured manifest of the OAS source files included in the study, together with the available source- and study-level metadata, including OAS dataset name, sequence count, organism, chain type, isotype, disease and vaccination annotations, individual identifier where available, age, longitudinal annotation, and mapping to the six-IGHV analyses. The same workbook summarizes metadata completeness and source-file coverage. PairedAbNGS data used for the external-resource analyses were obtained from the public PairedAbNGS FASTA release described by Dudzic et al. (54). The corresponding source accessions, parsing and quality-control summaries, and identifiable overlap with OAS are reported in the **Supplementary Materials**. Derived results generated in this study are provided in the **Supplementary Materials**. No new experimental sequencing data were generated in this study. The preprocessing and analysis workflow is documented in the accompanying reproducibility package, including README_reproducibility_workflow_R2.md, run_r2_reproducibility.py, run_r2_reproducibility.sh, the analysis scripts, environment specifications, and input/output manifests.
