# Supplementary material for: Recurrent antibody repertoire features associated with six selected human IGHV genes: V(D)J usage, heavy-light pairing, and CDRH3 properties
Source: Front Immunol. 2026 Sep 21;17:1888170. doi: 10.3389/fimmu.2026.1888170 (PMC13635128; doi:10.3389/fimmu.2026.1888170)
Supplement: Supplementary file 1 [file SupplementaryFile1.docx]

## **Supplementary material**

**Supplementary Figures Legend**

Supplementary Figure S1. Comprehensive landscape of gene usage and pairing preferences in the human paired antibody repertoire. Circos and Sankey visualizations summarize global IGHV-light-chain and V(D)J usage patterns, with the six selected IGHV genes highlighted.

Supplementary Figure S2. Complete Sankey rearrangement maps of the six selected IGHV genes. Separate panels show V(D)J usage trajectories for IGHV1-69, IGHV1-2, IGHV3-21, IGHV3-23, IGHV3-30, and IGHV4-34.

Supplementary Figure S3. Readable summary of dominant V(D)J and light-chain V-gene usage across the six focus IGHV backgrounds.

Supplementary Figure S4. Conditional D-J pairing frequencies across IGHV backgrounds. Heatmaps show observed D-J pairing frequencies within each of the six selected IGHV backgrounds.

Supplementary Figure S5. Conditional D-J enrichment and depletion across IGHV backgrounds. Standardized residual heatmaps show enrichment and depletion of D-J combinations after accounting for marginal D- and J-gene usage within each IGHV background.

Supplementary Figure S6. Immune-context composition of the paired OAS collection. (A) Source-record counts assigned to eight metadata-defined immune-context categories. (B) Percentage of records from sources with an explicit disease and/or vaccine annotation versus unclassified or unannotated sources.

Supplementary Figure S7. Chord diagrams of VH-VL pairing networks. Chord diagrams display the top light-chain partners for each of the six selected IGHV genes, emphasizing prominent kappa and lambda pairing patterns.

Supplementary Figure S8. Effect-size-first bootstrap support for representative associations. Forest-style plot showing log2 observed/expected values with study-level bootstrap 95% confidence intervals for representative VH-VL, V-D, and V-conditioned D-J associations.

Supplementary Figure S9. Key VH-VL associations across metadata-defined immune contexts. Heatmap showing log2(O/E) for the 11 prespecified pairs after deduplication within study-context strata.

Supplementary Figure S10. VH-conditioned light-chain concentration and shared-heavy light-chain coherence proxy. (A) Simpson light-chain V-gene concentration across study-VH units. (B) Observed cross-donor light-chain V-gene agreement for recurrent exact shared-heavy clonotypes and the corresponding within-study VH-marginal baseline.

Supplementary Figure S11. Conservation of light-chain V-J usage across heavy-chain backgrounds. Heatmaps summarize light-chain V-J usage when paired with different heavy-chain V genes.

Supplementary Figure S12. CDRH3 amino acid usage fingerprints and composition profiles associated with selected IGHV genes. Radar and lollipop plots show IGHV-associated amino acid composition and enrichment/depletion patterns in CDRH3 regions.

Supplementary Figure S13. IGHV-associated CDRH3 terminal-position amino-acid profiles. Heatmaps show amino-acid frequencies at the first five N-terminal and last five C-terminal positions of CDRH3 for each of the six selected IGHV backgrounds.

Supplementary Figure S14. Sensitivity of PairedAbNGS VH-VL associations to identifiable OAS overlap. (A) Identifiable overlap between OAS and PairedAbNGS at the run-accession, sequence-row, and exact-clonotype levels. (B) PairedAbNGS direct log2(O/E) values before and after exclusion of records with identifiable overlap. (C) Pair-level changes in direct log2(O/E) after overlap exclusion. Direct effects were calculated only for positive observed and expected counts.

Supplementary Figure S15. Cross-resource comparison of IGHV-associated CDRH3 physicochemical features. Median and interquartile range of CDRH3 amino-acid length, simplified side-chain charge score, and Kyte-Doolittle GRAVY hydropathy in OAS and PairedAbNGS.

Supplementary Figure S16. V-conditioned D-J pairing frequencies in PairedAbNGS. Heatmaps show observed conditional D-J frequencies within each of the six focus IGHV backgrounds for standardized D calls shared with the OAS analysis.

Supplementary Figure S17. V-conditioned D-J direct effect sizes in PairedAbNGS. Heatmaps show conditional D-J direct log2(O/E) values after accounting for D- and J-gene marginals within each IGHV background. Values were calculated as log2(Observed/Expected) for cells with positive observed and expected counts. Numeric annotations show supported cells.

Supplementary Figure S18. Cross-resource concordance of V-conditioned D-J associations. (A) OAS and PairedAbNGS direct conditional D-J effects for cells meeting support thresholds in both resources. (B) Directional concordance in PairedAbNGS for prespecified OAS study-robust D-J calls. (C) Sensitivity to removal of records with identifiable OAS overlap. All effect sizes use direct log2(Observed/Expected) for positive observed and expected counts; no pseudocount was used.

Supplementary Figure S19. Study-aware analysis of the IGHV4-34-associated CDRH3 side-chain charge-score shift in OAS. Points show study-level mean-score differences between IGHV4-34 and the other five focus IGHV backgrounds; the summary shows the random-effects estimate and Hartung-Knapp 95% confidence interval.

Supplementary Figure S20. Immune-context sensitivity of the IGHV4-34-associated CDRH3 side-chain charge score shift. Filled estimates denote contexts supported by at least three eligible studies; categories with fewer studies are shown descriptively. Unclassified or unannotated samples were not treated as healthy naïve repertoires.

**Supplementary Table Legends**

Supplementary Table S1. Structured OAS source-file metadata. Per-source and per-study metadata table listing the 563 OAS source files mapped to 17 validated DS Name/source-study groups for metadata-linked and study-aware analyses. Columns include OAS source file, DS Name, sequence count, organism, chain type, isotype, disease annotation, vaccine annotation, individual identifier where available, age, longitudinal annotation, and source-file mapping. The native OAS download comprised 580 source files from 19 OAS URL study directories; 17 source files without validated structured metadata mapping were retained only for native-source productivity/schema auditing and were not used as study-resampling units.

Supplementary Table S2. Observed and expected counts, direct log2 observed-to-expected ratios [log2(O/E)] for cells with positive observed and expected counts, 95% confidence intervals from 1,000 study-level bootstrap replicates, threshold indicators, and study-level directional concordance for VH-VL, V-D, and V-conditioned D-J associations among the six selected IGHV backgrounds.

Supplementary Table S3. Availability of productive and non-productive rearrangements in the OAS source data.

Supplementary Table S4. Repertoire characteristics of all abundant IGHV genes. Summary of the 22 IGHV genes represented by at least 50,000 valid, standardized IGHV-assigned sequence records. For each IGHV gene, the table reports record abundance, the most frequent D genes, light-chain V genes, and D-J combinations, CDRH3 amino-acid length summaries, and inclusion in the six selected IGHV set.

Supplementary Table S5. Immune-context composition, VH-VL sensitivity, and exploratory light-chain coherence analyses. Characterization of disease and vaccination metadata in the paired OAS datasets, together with key VH-VL effects stratified by immune context and contributing study.

Supplementary Table S6. Observed and expected counts, observed-to-expected ratios, direct log2(O/E) values for cells with positive observed and expected counts, odds ratios, and FDR-adjusted P values for 395 VH-VL combinations among the six selected IGHV backgrounds.

Supplementary Table S7. Sensitivity to 85%-identity CDRH3 clustering. Comparison of exact-clonotype and 85%-identity CDRH3-cluster analyses for 11 key VH-VL pairs, 40 V-D effects, and 120 V-conditioned D-J effects. The table reports observed counts, expected counts, log2(O/E) effect sizes, enrichment/depletion directions, direction concordance, delta log2(O/E), and summary statistics for effect-size concordance.

Supplementary Table S8. IGHV-specific empirical ranges of CDRH3 sequence features. CDRH3 amino-acid length, simplified side-chain charge score, and Kyte-Doolittle grand average of hydropathy (GRAVY) summarized for each of the six selected IGHV genes.

Supplementary Table S9. Descriptive CDRH3 sequence-based proxy across IGHV backgrounds. Sequence counts and summary statistics for absolute side-chain charge score, GRAVY, hydrophobic-residue fraction, liability-motif count, and the descriptive sequence-based proxy for each selected IGHV gene.

Supplementary Table S10. Sensitivity of CDRH3 side-chain charge estimates to the calculation method. Comparison of the simplified side-chain charge score with a pKa-based side-chain charge calculation at pH 7.4.

Supplementary Table S11. Field audit and discrete reconstruction of the CDRH3 length distribution.

Supplementary Table S12. Cross-resource comparison of key VH-VL associations and CDRH3 features. The workbook reports OAS and PairedAbNGS observed counts, expected counts, direct log2(O/E) values, association directions, direction concordance, and overlap-exclusion sensitivity for the 11 prespecified VH-VL pairs, together with IGHV-associated CDRH3 length, simplified side-chain charge-score, and GRAVY hydropathy summaries. Direct VH-VL effects were calculated only for positive observed and expected counts; no pseudocount was used.

Supplementary Table S13. Cross-resource comparison of V-conditioned D-J associations. The workbook reports OAS and PairedAbNGS observed counts, expected counts, conditional frequencies, direct log2(O/E) values, support indicators, direction concordance, and overlap-exclusion sensitivity for 792 V-conditioned D-J cells across the six focus IGHV backgrounds. Direct effects were calculated only for positive observed and expected counts; no pseudocount was used.

Supplementary Table S14. Study-aware and cross-resource analysis of the IGHV4-34-associated CDRH3 side-chain charge-score shift.
