# Supplementary material for: Recurrent antibody repertoire features associated with six selected human IGHV genes: V(D)J usage, heavy-light pairing, and CDRH3 properties
Source: Front Immunol. 2026 Sep 21;17:1888170. doi: 10.3389/fimmu.2026.1888170 (PMC13635128; doi:10.3389/fimmu.2026.1888170)
Supplement: Supplementary file 2 [file SupplementaryFile2.zip › supplementary_figures/FigureS11.pdf]

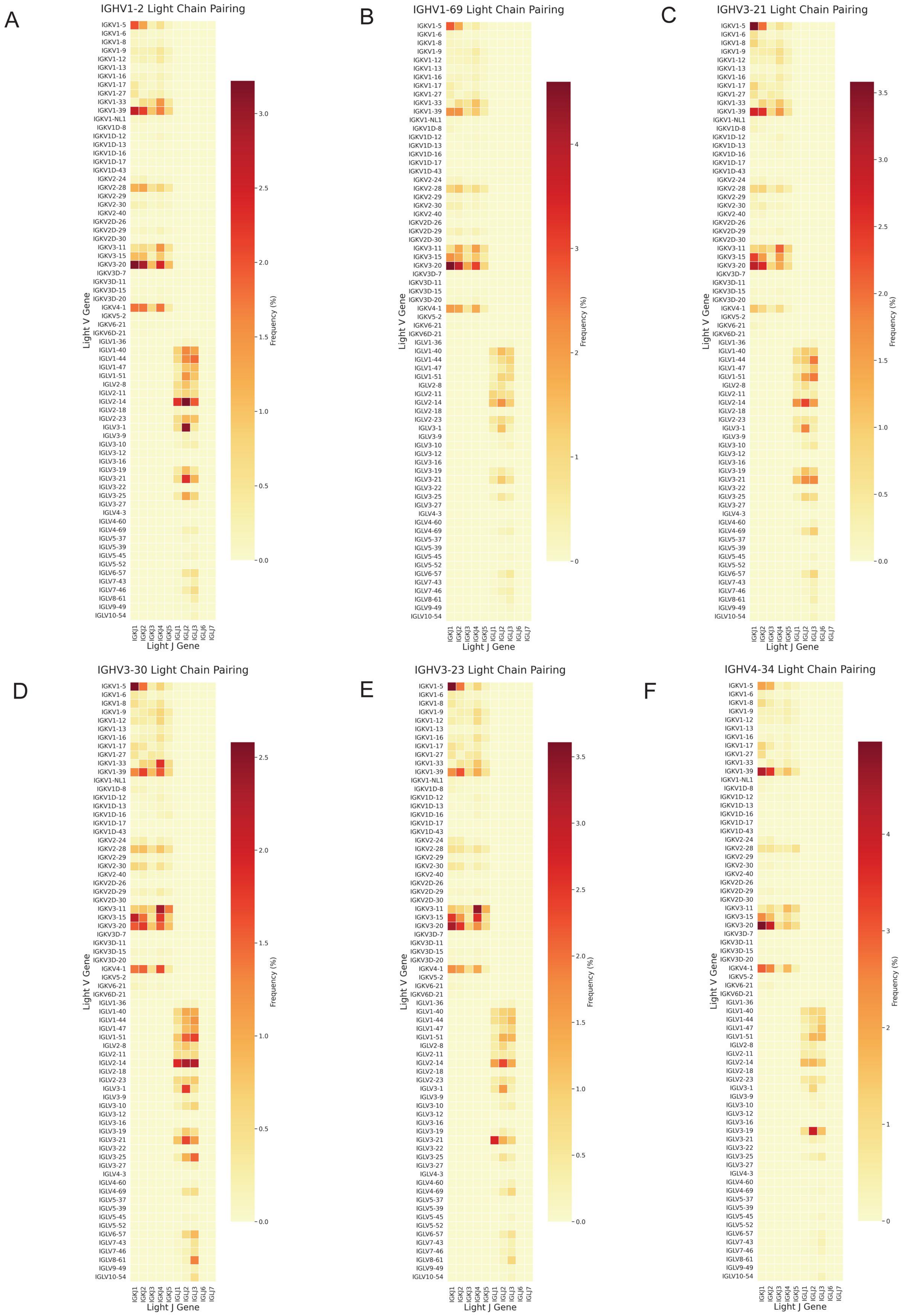
A
IGHV1-2 Light Chain Pairing
B
IGHV1-69 Light Chain Pairing
C
IGHV3-21 Light Chain Pairing
D
IGHV3-30 Light Chain Pairing
E
IGHV3-23 Light Chain Pairing
F
IGHV4-34 Light Chain Pairing
Light V Gene
Light J Gene
Frequency (%)
IGKV1-5
IGKV1-6
IGKV1-8
IGKV1-9
IGKV1-12
IGKV1-13
IGKV1-16
IGKV1-17
IGKV1-27
IGKV1-33
IGKV1-39
IGKV1-NL1
IGKV1D-8
IGKV1D-12
IGKV1D-13
IGKV1D-16
IGKV1D-17
IGKV1D-43
IGKV2-24
IGKV2-28
IGKV2-29
IGKV2-30
IGKV2-40
IGKV2D-26
IGKV2D-29
IGKV2D-30
IGKV3-11
IGKV3-15
IGKV3-20
IGKV3D-7
IGKV3D-11
IGKV3D-15
IGKV3D-20
IGKV4-1
IGKV5-2
IGKV6-21
IGKV6D-21
IGLV1-36
IGLV1-40
IGLV1-44
IGLV1-47
IGLV1-51
IGLV2-8
IGLV2-11
IGLV2-14
IGLV2-18
IGLV2-23
IGLV3-1
IGLV3-9
IGLV3-10
IGLV3-12
IGLV3-16
IGLV3-19
IGLV3-21
IGLV3-22
IGLV3-25
IGLV3-27
IGLV4-3
IGLV4-60
IGLV4-69
IGLV5-37
IGLV5-39
IGLV5-45
IGLV5-52
IGLV6-57
IGLV7-43
IGLV7-46
IGLV8-61
IGLV9-49
IGLV10-54
IGKJ1
IGKJ2
IGKJ3
IGKJ4
IGKJ5
IGLJ1
IGLJ2
IGLJ3
IGLJ6
IGLJ7
