# Supplementary material for: Recurrent antibody repertoire features associated with six selected human IGHV genes: V(D)J usage, heavy-light pairing, and CDRH3 properties
Source: Front Immunol. 2026 Sep 21;17:1888170. doi: 10.3389/fimmu.2026.1888170 (PMC13635128; doi:10.3389/fimmu.2026.1888170)
Supplement: Supplementary file 2 [file SupplementaryFile2.zip › supplementary_figures/FigureS13.pdf]

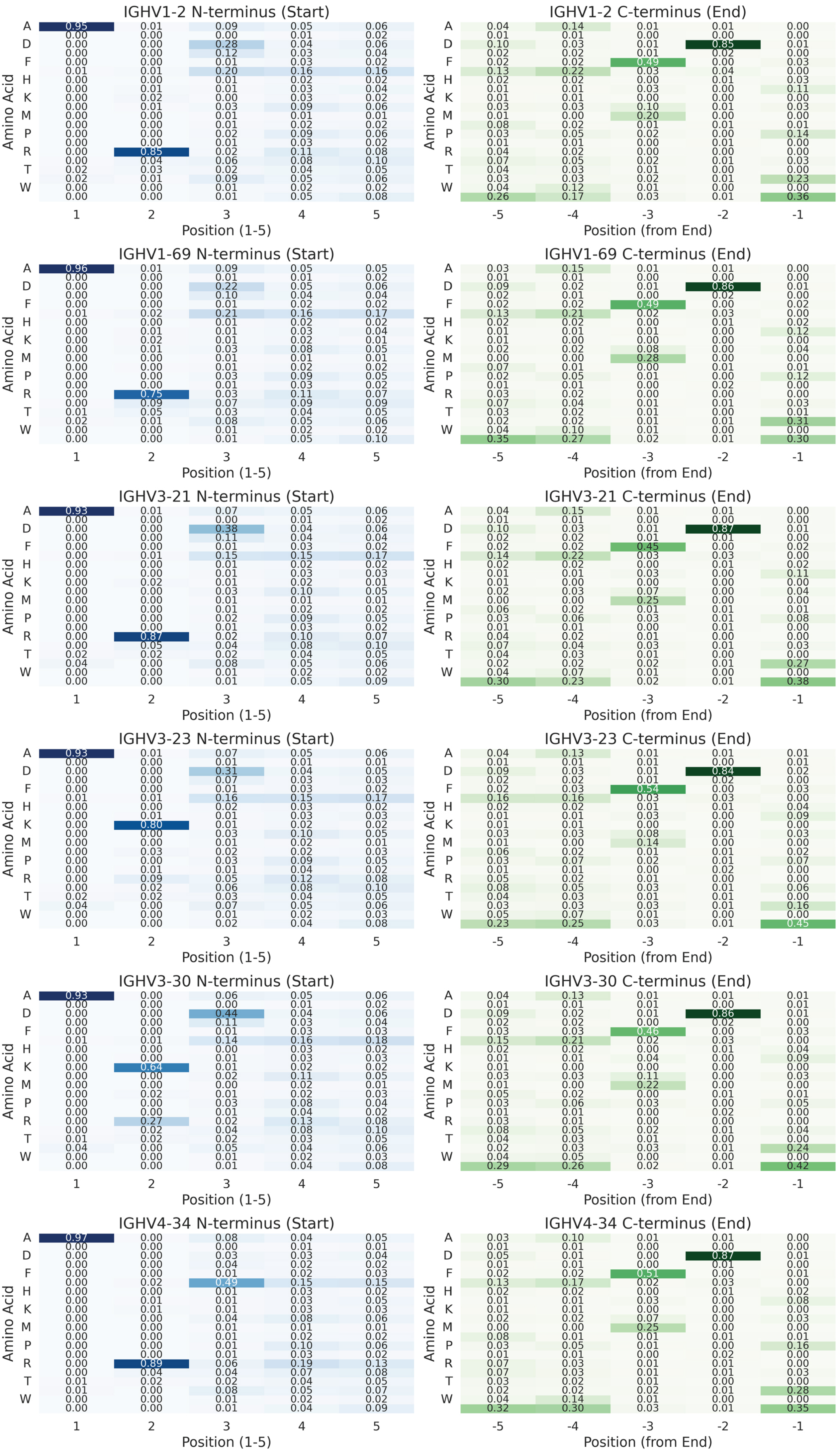
IGHV1-2 N-terminus (Start)
IGHV1-2 C-terminus (End)
IGHV1-69 N-terminus (Start)
IGHV1-69 C-terminus (End)
IGHV3-21 N-terminus (Start)
IGHV3-21 C-terminus (End)
IGHV3-23 N-terminus (Start)
IGHV3-23 C-terminus (End)
IGHV3-30 N-terminus (Start)
IGHV3-30 C-terminus (End)
IGHV4-34 N-terminus (Start)
IGHV4-34 C-terminus (End)
Amino Acid
Position (1-5)
Position (from End)
