# Supplementary material for: Recurrent antibody repertoire features associated with six selected human IGHV genes: V(D)J usage, heavy-light pairing, and CDRH3 properties
Source: Front Immunol. 2026 Sep 21;17:1888170. doi: 10.3389/fimmu.2026.1888170 (PMC13635128; doi:10.3389/fimmu.2026.1888170)
Supplement: Supplementary file 2 [file SupplementaryFile2.zip › supplementary_figures/FigureS16.pdf]

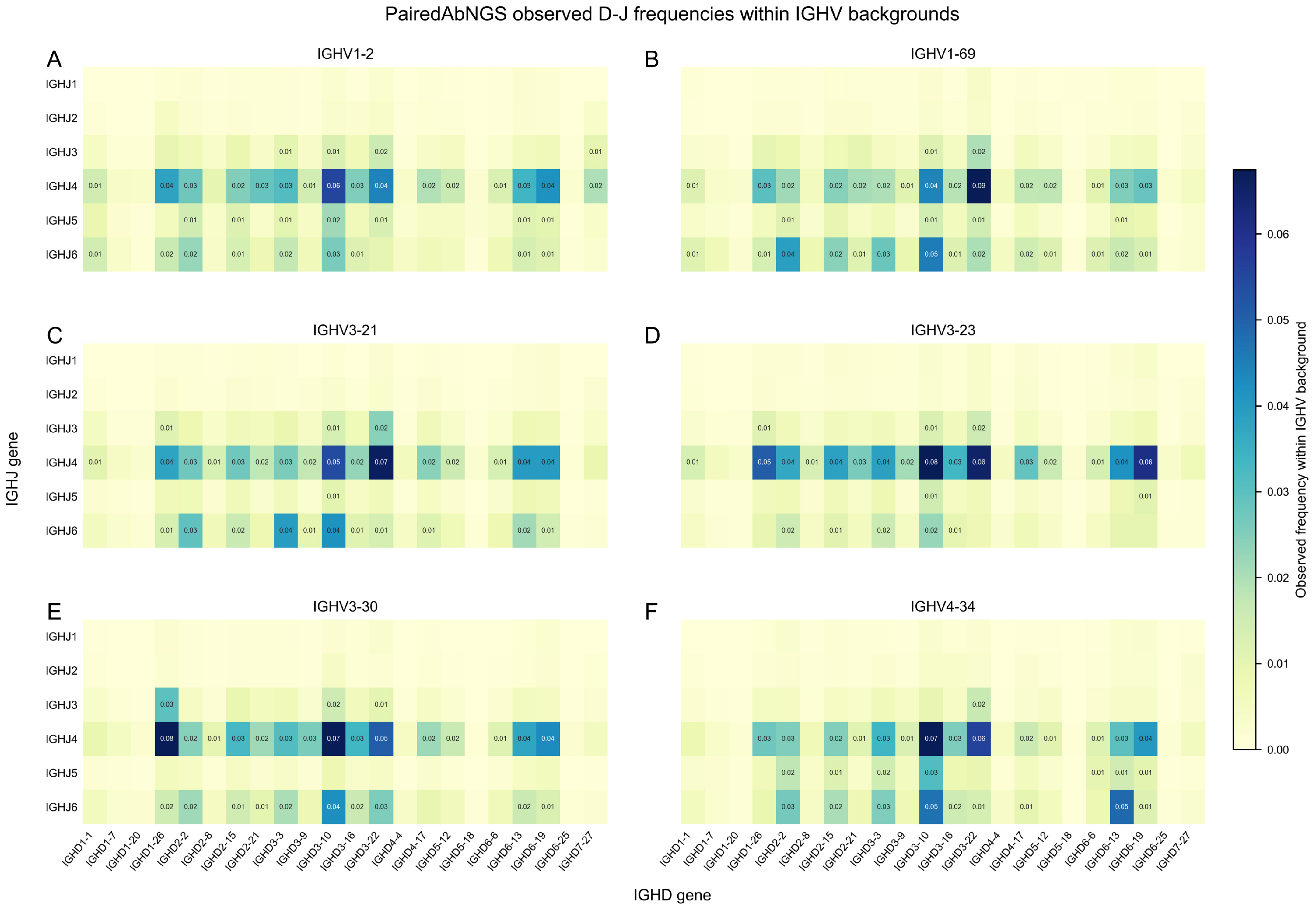
PairedAbNGS observed D-J frequencies within IGHV backgrounds
A
IGHV1-2
B
IGHV1-69
C
IGHV3-21
D
IGHV3-23
E
IGHV3-30
F
IGHV4-34
IGHJ1
IGHJ2
IGHJ3
IGHJ4
IGHJ5
IGHJ6
IGHJ gene
IGHD1-1
IGHD1-7
IGHD1-20
IGHD1-26
IGHD2-2
IGHD2-8
IGHD2-15
IGHD2-21
IGHD3-3
IGHD3-9
IGHD3-10
IGHD3-16
IGHD3-22
IGHD4-4
IGHD4-17
IGHD5-12
IGHD5-18
IGHD6-6
IGHD6-13
IGHD6-19
IGHD6-25
IGHD7-27
IGHD gene
0.00
0.01
0.02
0.03
0.04
0.05
0.06
Observed frequency within IGHV background
