# Supplementary material for: Recurrent antibody repertoire features associated with six selected human IGHV genes: V(D)J usage, heavy-light pairing, and CDRH3 properties
Source: Front Immunol. 2026 Sep 21;17:1888170. doi: 10.3389/fimmu.2026.1888170 (PMC13635128; doi:10.3389/fimmu.2026.1888170)
Supplement: Supplementary file 2 [file SupplementaryFile2.zip › supplementary_figures/FigureS2.pdf]

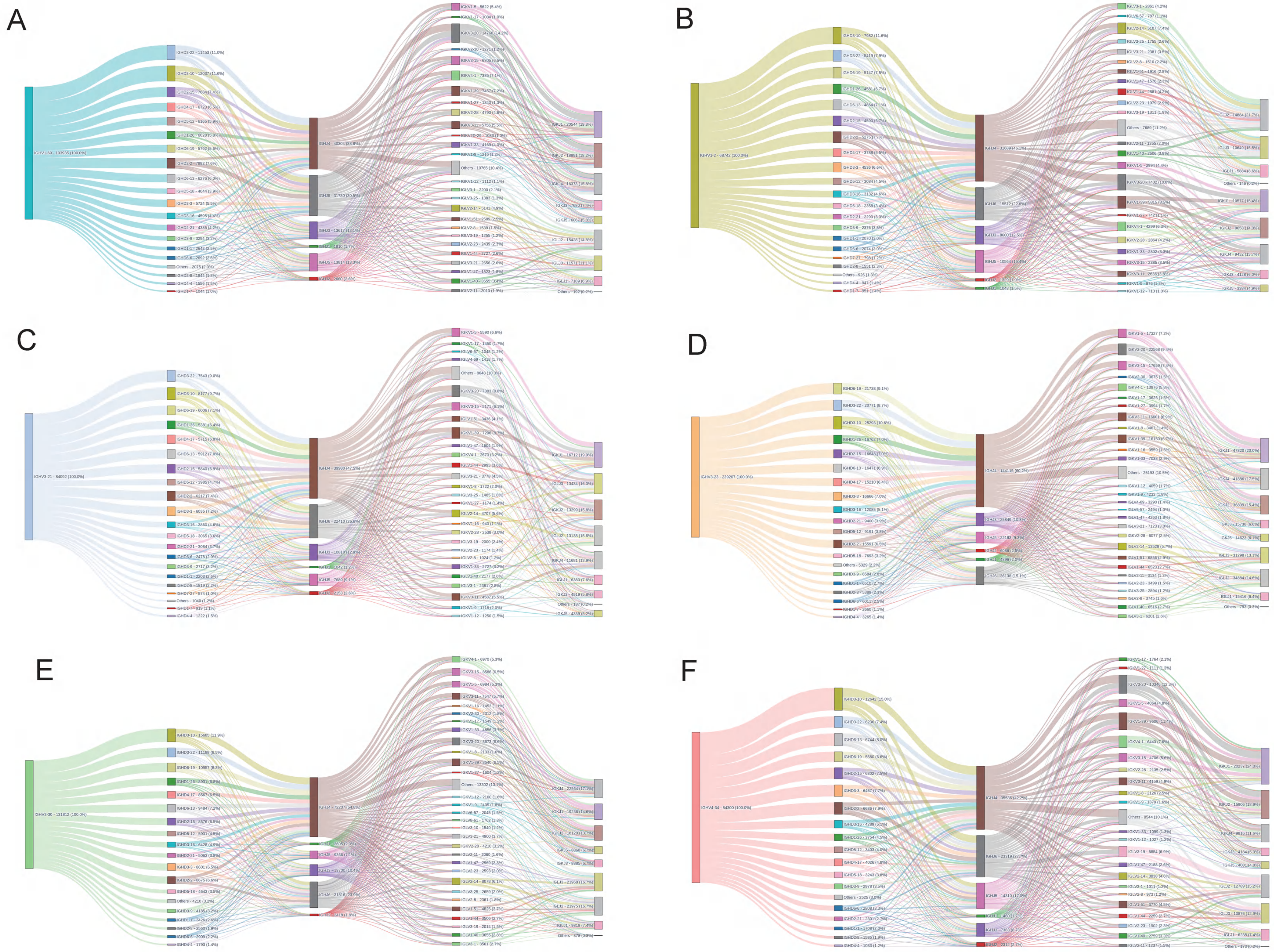
A
B
C
D
E
F
