# Supplementary material for: Recurrent antibody repertoire features associated with six selected human IGHV genes: V(D)J usage, heavy-light pairing, and CDRH3 properties
Source: Front Immunol. 2026 Sep 21;17:1888170. doi: 10.3389/fimmu.2026.1888170 (PMC13635128; doi:10.3389/fimmu.2026.1888170)
Supplement: Supplementary file 2 [file SupplementaryFile2.zip › supplementary_figures/FigureS7.pdf]

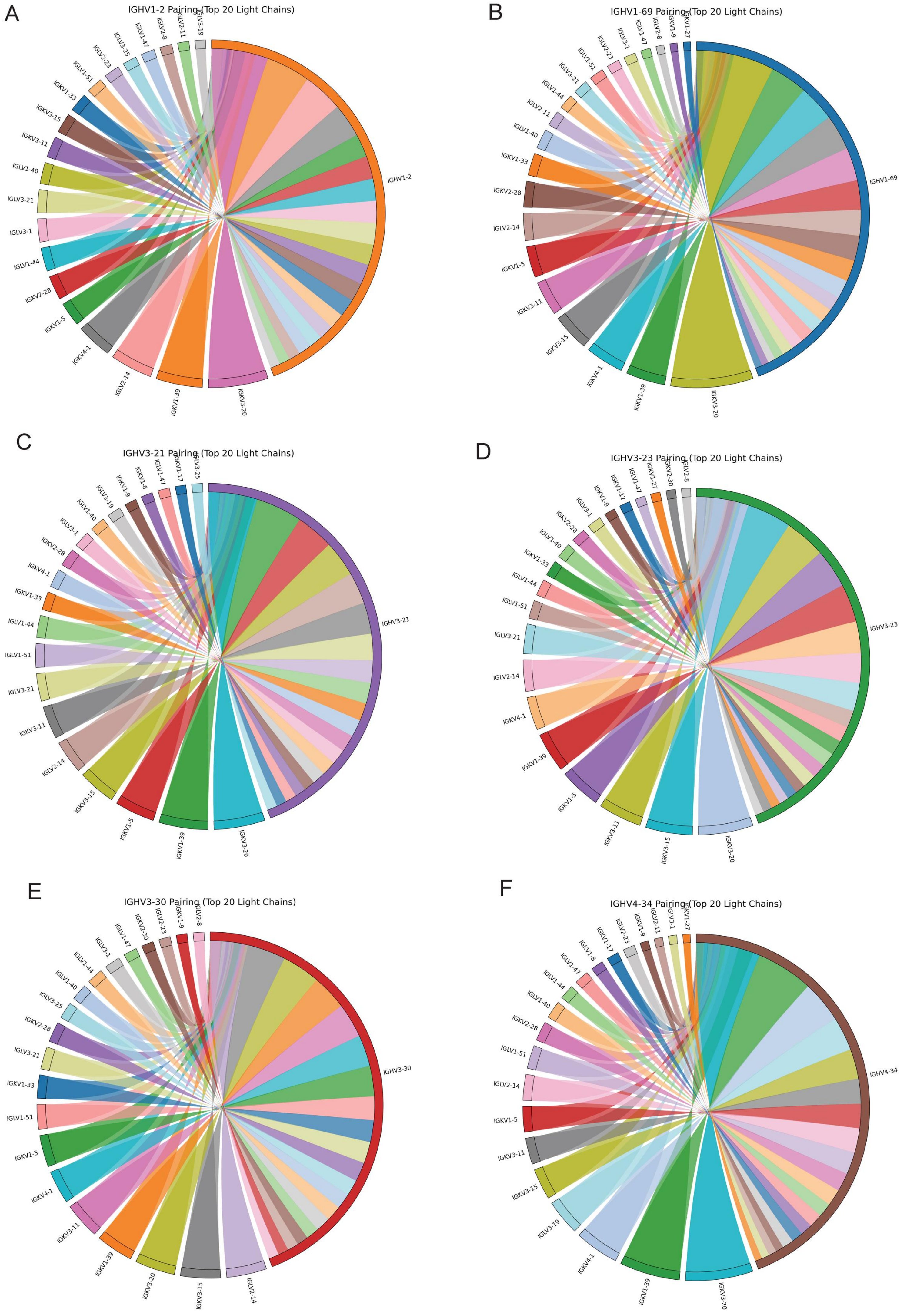
A
IGHV1-2 Pairing (Top 20 Light Chains)
IGLV3-19
IGLV2-11
IGLV2-8
IGLV1-47
IGLV3-25
IGLV2-23
IGLV1-51
IGKV1-33
IGKV3-15
IGKV3-11
IGLV1-40
IGLV3-21
IGLV3-1
IGLV1-44
IGKV2-28
IGKV1-5
IGKV4-1
IGLV2-14
IGKV1-39
IGKV3-20
IGHV1-2
B
IGHV1-69 Pairing (Top 20 Light Chains)
IGKV1-27
IGKV1-9
IGLV2-8
IGLV1-47
IGLV3-1
IGLV2-23
IGLV1-51
IGLV3-21
IGLV1-44
IGLV2-11
IGLV1-40
IGKV1-33
IGKV2-28
IGLV2-14
IGKV1-5
IGKV3-11
IGKV3-15
IGKV4-1
IGKV1-39
IGKV3-20
IGHV1-69
C
IGHV3-21 Pairing (Top 20 Light Chains)
IGLV3-25
IGKV1-17
IGLV1-47
IGKV1-8
IGKV1-9
IGLV3-19
IGLV1-40
IGLV3-1
IGKV2-28
IGKV4-1
IGKV1-33
IGLV1-44
IGLV1-51
IGLV3-21
IGKV3-11
IGLV2-14
IGKV3-15
IGKV1-5
IGKV1-39
IGKV3-20
IGHV3-21
D
IGHV3-23 Pairing (Top 20 Light Chains)
IGLV2-8
IGKV2-30
IGKV1-27
IGLV1-47
IGKV1-12
IGKV1-9
IGLV3-1
IGKV2-28
IGLV1-40
IGKV1-33
IGLV1-44
IGLV1-51
IGLV3-21
IGLV2-14
IGKV4-1
IGKV1-39
IGKV1-5
IGKV3-11
IGKV3-15
IGKV3-20
IGHV3-23
E
IGHV3-30 Pairing (Top 20 Light Chains)
IGLV2-8
IGKV1-9
IGLV2-23
IGKV2-30
IGLV1-47
IGLV3-1
IGLV1-44
IGLV1-40
IGLV3-25
IGKV2-28
IGLV3-21
IGKV1-33
IGLV1-51
IGKV1-5
IGKV4-1
IGKV3-11
IGKV1-39
IGKV3-20
IGKV3-15
IGLV2-14
IGHV3-30
F
IGHV4-34 Pairing (Top 20 Light Chains)
IGKV1-27
IGLV3-1
IGLV2-11
IGKV1-9
IGLV2-23
IGKV1-17
IGKV1-8
IGLV1-47
IGLV1-44
IGLV1-40
IGKV2-28
IGLV1-51
IGLV2-14
IGKV1-5
IGKV3-11
IGKV3-15
IGLV3-19
IGKV4-1
IGKV1-39
IGKV3-20
IGHV4-34
